# Climate change and land-use change impacts on future availability of forage grass species for Ethiopian dairy systems

**DOI:** 10.1038/s41598-022-23461-w

**Published:** 2022-11-28

**Authors:** Galina Brychkova, Kelebogile Kekae, Peter C. McKeown, Jean Hanson, Chris S. Jones, Philip Thornton, Charles Spillane

**Affiliations:** 1 Agriculture and Bioeconomy Research Centre, Ryan Institute, University of Galway, University Road, Galway, H91 REW4 Ireland; 2 CGIAR Research Program On Climate Change, Agriculture and Food Security (CCAFS), International Livestock Research Institute (ILRI), Nairobi, Kenya

**Keywords:** Plant sciences, Climate sciences, Climate change

## Abstract

Forage grasses are central feed resources for livestock globally. In Ethiopian dairy systems, they serve as feed sources during both wet and dry seasons, yet escalating climate change could threaten forage supply. Here, we investigate projected climate change impacts on three forage grasses currently recommended for Ethiopian dairy systems. We determine areas of geographical suitability for each species using three climate projections generated by General Circulation Models (GCMs) and calculate their ability to meet predicted dry matter demand under four scenarios for livestock intensification and land availability. By 2050, Buffel grass (*Cenchrus ciliaris*) is likely to be negatively affected by climate change in regions such as Tigray, while Rhodes grass (*Chloris gayana*) and Napier grass (*Cenchrus purpureus*) may have improved suitability under future climates. Our findings suggest that feed demands could theoretically be met by production of these forage grasses under current and future climates. However, if land availability is reduced and herd composition shifts towards higher-productivity exotic breeds, forage resources will not meet cattle demand even with improved agronomic management.

## Introduction

Livestock systems play vital roles in sustaining livelihoods in many African countries. Over 80% of Ethiopia’s 82 m people are agriculture-dependent^1,2^: Ethiopia maintains herds of ~ 55.6 m cattle, 24.1 m goats, 25.4 m sheep and 0.91 m camels, plus equids^3–5^. Livestock production is increasing throughout Sub-Saharan Africa, driven by population growth, urbanization and rising living standards which increase demand for livestock products^6^ and therefore demand for feed. In 2018, feed needs for Ethiopia were ~ 130 m tons with a negative absolute feed balance^7^. The Ethiopian government’s livestock master plan highlights that, absent interventions to improve feed resources, future feed demand cannot be met^8^. Indeed, land availability decreased by 18.1% from 1973 to 2012, as natural pasture was replaced with crop lands^9–11^. In 2015, only 15.2% of Ethiopia’s area was arable, including meadows, pasture, market and kitchen gardens^12^. As a result, most Ethiopian dairy farmers practice cut-and-carry systems without grazing, with only a few combining the systems^8^.

To benefit from risk-reduced pastoral strategies, sufficient feed of suitable quality should be available^13^. In Ethiopian regions such as Somali, Benishangul-Gemuz and Gambela, grazing feed accounts for 117%, 197% and 373% of annual feed requirements, respectively, resulting in a positive absolute feed balance while in areas with negative feed balance, grazing feed contributes only 31–46%^7^. The best grass varieties for supporting the country’s future livestock production in a potentially sustainable manner must be chosen^14–18^. Amongst those recommended for improved management practices, three forage grass species, commonly called Buffel (*Cenchrus ciliaris*), Napier (formerly *Pennisetum purpureum,* now *Cenchrus purpureus*), and Rhodes (*Chloris gayana*) grasses are most common^15,19^. All are perennial C4 grasses whose growth and yield are expected to be positively affected by rising ambient CO_2_ (Table 1). Currently, both Napier and Rhodes grasses are used by Ethiopian smallholders for cattle fattening and are associated with higher milk yields^9^. Farmers typically use these forages interchangeably in zero-grazing systems to produce hay or silage^20,21^.

**Table 1:** Traits of Buffel, Napier and Rhodes grass grown under conditions currently prevalent in sub-Saharan Africa^20–29^.

	Buffel grass	Napier grass	Rhodes grass
**General traits**			
Drought tolerant	Yes	Moderate	Yes
Tolerates saline soils	No	No	Yes
Sunlight species	No	Yes	Yes
Tolerates water logging	No	No	Seasonal water logging
Suitability for hay	Yes	Yes	Yes
Suitability for silage	No	Yes	No
Forage system	Grazing	Zero grazing	Grazing and Zero grazing
Yield increase when grown with other species i.e. legumes	No	Yes	Yes
Yield without fertilizers, Ethiopia (t/ha/year)	2–9 DM	2–9 DM	2–7 DM
Max yield (Ethiopia), (t/ha/year)	9–16 DM	9–30 DM	7–18 DM
**Ruminant nutritive values**			
Crude protein (CP, %)	6–10	7.5–15.7	4.4–16.6
OM digestibility, ruminants (%)	56.7	61.4	60.4
Energy digestibility, ruminants (%)	54.2	58.7	57.7
DE ruminants (MJ/kg DM)	9.9	10.2	19.6
Neutral detergent fibre (NDF, %)	63.1–75.1	52–64.6	70.5–80.8
Acid detergent fibre (ADF%)	33.2–46.6	28.8–36.6	37–50.1
**Minerals**			
Manganese (g/kg DM)	33	91	72
Zinc (g/kg DM)	179	45	28
Copper (g/kg DM)	14	11	6
Iron (g/kg DM)	1687	413	237

Buffel grass is considered one of the most productive forages for semi-arid areas in the subtropics and tropics^21^ and is cultivated in permanent pastures and leys^22^. Regarded as drought-tolerant, it grows naturally in areas with annual rainfall as low as 270 mm, up to 3500 mm, but cannot withstand prolonged waterlogging. Its optimal best-performance day temperatures are 30–35 °C^23,24^ (Table 1).

Napier grass (or elephant grass) is a tall, fast-growing summer grass^25^ which withstands repeated cutting as it regenerates rapidly, producing a high biomass that is very palatable in the leafy stage^21^. It performs well in temperatures of 21–40 °C^22^ and with annual rainfall > 1000 mm^21^. Napier grass survives drought reasonably when established because of its deep roots, but prefers well-drained soils and does not tolerate flooding^21^. Fertilizer application produces yields^20,26^ of 20–80 t DM ha^−1^ yr^−1^, or 2–10 t DM ha^−1^ yr^−1^ without adequate fertilizer^27^.

Rhodes grass is a stoloniferous grass that makes good hay if cut as it begins to flower, but cannot be used for silage^28^. It grows in a wide range of soils with annual rainfall 600–1500 mm and temperatures 20–37 °C, and tolerates both drought and seasonal waterlogging. If cultivated in properly prepared land on moderately fertile soil, yield can be 7–18 DM ha^−1^ yr^−1^, depending on variety, environmental conditions and cutting frequency^21,29^.

Future climate projections indicate that temperatures will increase by between 2–6 °C by 2100, depending on region and emissions scenario^30,31^ and tropical rainforest climatic zone will expand substituting temperate zones in the study areas (Suppl. Fig. S1D). Ethiopia is particularly vulnerable to climate change due to its significant reliance on agriculture, low adaptive capacity, geographical location, and social and economic structure^32–34^.

The physiological impacts of climate change on pasture crops are wide-ranging and highly dependent upon genotype and the nature of stress^35,36^. A study integrating temperature and rainfall predicted that length of growing period (LGP) for many crops will reduce across much of Sub-Saharan Africa, sometimes severely^37^. Rising CO_2_ levels can also alter herbage quality by modulating concentrations of water-soluble carbohydrates and N^38^. Additionally, high temperature during critical growth periods e.g. establishment or flowering, may lower CO_2_ effects on yield^39^. Moreover, the frequencies of extreme events, including heatwaves, drought and flooding, will have further adverse effects on crop and livestock productivity^40^. Most of Ethiopia experiences one main wet season from mid‐June to mid-September with < 350 mm monthly rainfall in the wettest regions, but the onset and duration of rainfall varies considerably inter-annually causing frequent drought^41^. Hence, climate impacts on food security may vary significantly from area to area^42,43^.

**Figure 1:**
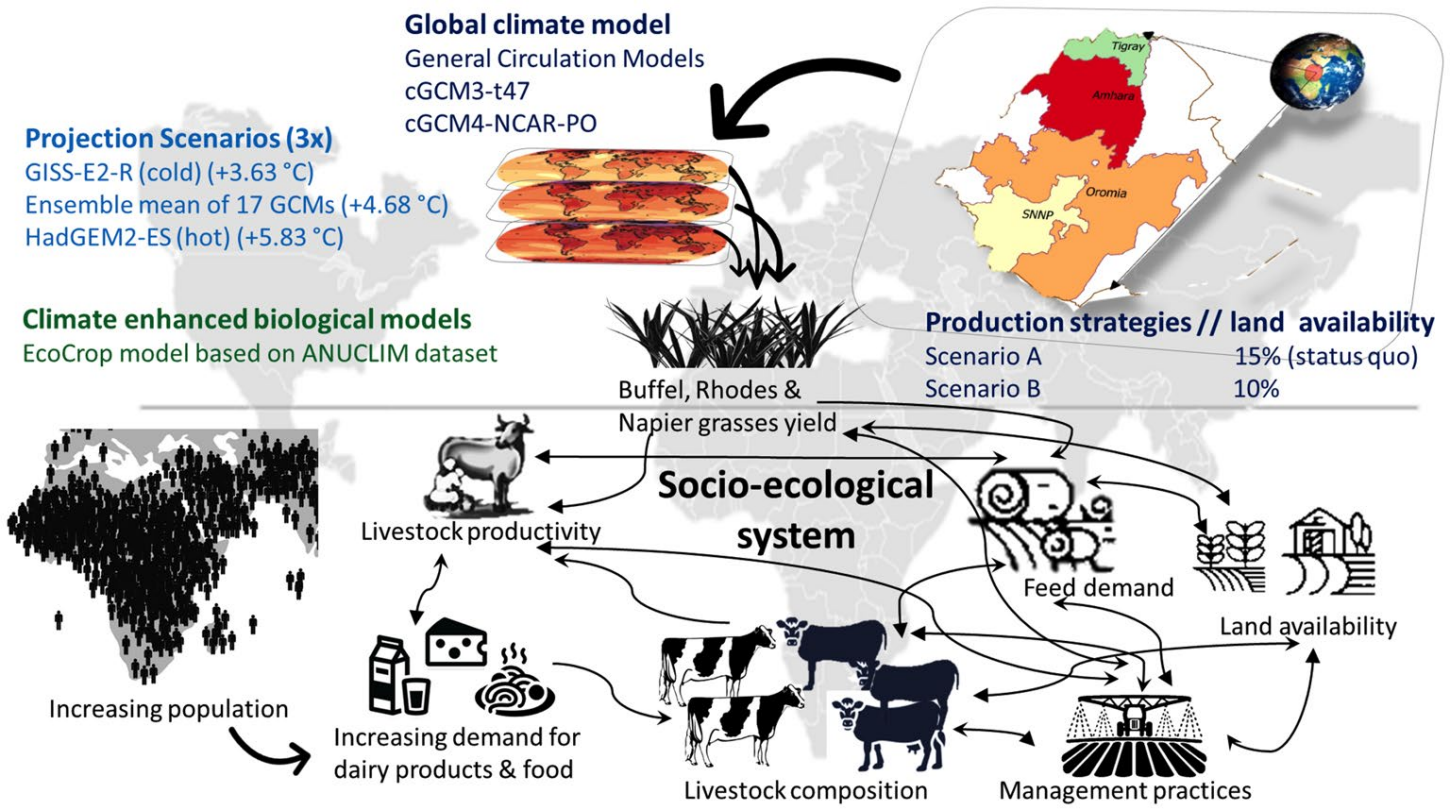
Elements of integrated modelling for evaluating climate impacts in 2050 on Buffel, Rhodes and Napier Grass contributions to cattle feed demand in Ethiopia as predicted by three future climate models.

To investigate the effect of climate change on Ethiopian forage production, the interactions between precipitation, temperature and land availability must be considered simultaneously. Here, we simulate growing parameters for Rhodes, Napier and Buffel grasses to address the bioclimatic suitability of each, defined by plant physiological responses to ecosystem constraints^44^. To assess the main model- and climate-driven uncertainties, and present a plausible range of response to climate impact, we compared three 2050 climatic models for the high GHG emissions scenario RCP 8.5. We combine projected climate impacts to the grasses with scenarios modelling future land availability and forage demand to explain the interactive effect between climate change and socio-economic drivers (Fig. 1). Our findings can guide climate adaptation strategies for farmers and thereby reduce climate change impacts on livestock-producing households.

## Methods

### Description of the study sites

Ethiopia is the second most populous country in Africa and is heavily dependent upon rainfed agriculture and pastoralism. The country is divided into nine regions for administrative purposes, as well as two chartered cities. This study analysed four eastern regions of Ethiopia representing a range of latitudes from the northernmost region of Tigray to Amhara in the centre, Oromia in the south and centre and the region of the Southern Nations, Nationalities and Peoples (SNNP) in the south (Suppl. Fig. S1).

#### Tigray region

The landscape of Tigray is dominated by chains of mountains ranging from 3250 to 3500 m asl. The region has two altitude extremes: the Tekeze Gorge, 550 m asl and the Kisad Gudo peak at 3, 935 m asl. The climate is characterized as *Kolla* (semi-arid) 39%; *Woina dega* (subtropical) 49% and *Dega* (temperate) 12%^45^; the average rainfall is between 450–980 mm. The dominant soil texture type is sandy loam, and all areas are in the moderate runoff hydrological zone^46^ (Suppl. Fig. S1B).

#### Amhara region

The Amhara region is topographically divided into the northern and eastern highlands at > 1500 m asl and the western and southern lowlands between 500–1500 m asl. Areas above 2300 m asl fall within the *Dega* climatic zone, those between 1500–2300 m asl within the *Woina Dega* climatic zone, and areas below 1500 m asl fall within the *Kolla* or hot climatic zones^45^. The annual mean temperature for most parts of the region is 15–21 °C; average rainfall ranges from 850 to 1485 mm with the highest rainfall occurring during the summer season (mid-June and ends in early September) but rains also occur from March–May^47^. The dominant soil texture types are sandy loams and loam, and the Amhara region is in the high to very high runoff hydrological zone^46^ (Suppl. Fig. S1B).

#### Oromia region

A region with an altitude of less than 1500 m above sea level. Climatic types prevailing the Oromia region may be grouped into three major categories: the dry climate, tropical rainy climate and temperate rainy climate. The dry climate is characterized by poor sparse vegetation with annual mean temperature of 27–39 °C, and mean annual rainfall of less than 450 mm. The hot semi-arid climate mean temperature ranges from between 18–27 °C. It has a mean annual rainfall of 410–820 mm with noticeable variability from year to year. The temperate climate areas have a moderate temperature and ample precipitation ranging between 1200–2000 mm. Oromia has predominantly loam and clay soils, while sandy loam soil texture type will be less than 20% (Suppl. Fig. S1B) while Oromia region hydrological zone is heterogenic, where west region is in high to very high hydrological zone, while east region of Oromia is graduated from moderate to high runoff hydrological zones^46^ (Suppl. Fig. S1C).

#### Southern nations, nationalities, and people’s region (SNNP)

SNNP has an elevation of 367–4207 m above sea level. About 56% of the total area of the region is found < 1500 m elevation, and is mostly classed as very hot lowland (*Kolla*) while the remaining 44% is found in the temperate climatic zone^45^. The annual rainfall of the region ranges from 500 to 2200 mm, with intensity, duration and amount all increasing from south to northeast and northwest; mean annual temperature of the region ranges from 15 to 30 °C. The soil texture in this region is dominated by loam texture type and this region is mostly considered a high runoff hydrological zone^46^ (Suppl. Fig. S1B,C).

### Climate change-informed trajectories for livestock feed security in Ethiopia

To investigate future trajectories for livestock feed security in Ethiopia, we developed socio-ecological models for possible changes occurring by 2050 (Fig. 1, Suppl. Fig. S2). All models assumed the growth of certain Kaya Identify^48^ factors: a 53% population increase and GDP increase per capita, leading to increased demands for dairy products and meat. To combine predicted future changes in forage productivity in different regions of Ethiopia with other factors likely to impact output, our models include likely increase of exotic livestock number^4^, either as per “Scenario A”, where local cattle breeds dominates and more productive exotic breeds accounts only to 7%, based on the current trend of 0.1–0.15% exotic breed increase annually^3^; or “Scenario B”, where herds will comprise 60%:40% local:exotic breeds to meet increasing food demand (Fig. S2). To model the sustainability of the scenarios by 2050, we considered ongoing reduction in grassland area^11^, a predicted ~ 40% loss by 2050^49^. Currently, 15% of Ethiopia is grassland^12^, thus, we assume that in 2050 possible available grassland will fall to ~ 10%. Since emission scenarios are uncertain under various climate factors, and Coupled Model Intercomparison Project (CMIP6) allows for warming of > 3 °C by 2100 even if emissions stay within plausible ranges^50^, our models assumed mean temperature increases > 3 °C. General circulation models (GCM) were used to develop three climate projection scenarios for 2050: GISS-2E-R, where mean annual temperature increases by + 1.96 °C; HadGEM2-ES, with + 2.93 °C and an ensemble mean of 17 GCMs, with + 2.36 °C (Table S1). We also considered grass productivity response to management (Table 1) and focussed on the major forage-growing regions in which negative feed balance has been recorded^7^, that is, Tigray, Amhara, Oromia and the Southern Nations, Nationalities, and Peoples (SNNP) Region, spanning the geographical range of Ethiopia from North to South. Elements included in the integrated modelling are presented in Fig. 1.

### Climate modelling

For recent climatic conditions, we used the WorldClim dataset version 2.0, that provides average monthly climate data for recent conditions (approximately 1970–2000) in terms of average daily maximum and minimum temperatures for each month and average monthly rainfall, at 5 min of arc spatial resolution. To evaluate a range of possible future climatic conditions, we used the methods of^51,52^ to downscale spatially coarse output from different groupings of the IPCC’s CMIP5 set of General Circulation Models^53^ (GCMs) to 5 min of arc, generating grid cells of size ~ 9 by 9 km at the Equator^51,52^. To present a plausible range of responses, the three sets of GCM outputs (Suppl. Table S1) with a high GHG emissions scenario RCP 8.5 were used: the first was an ensemble mean of 17 GCMs, but following^54^ we also considered two groupings providing bounds on either side of this, namely a “colder” CGM derived from GISS-E2-R (NASA Goddard Institute for Space Studies) representing a < 2 °C global heating situation by 2050, and a “warmer” GCM derived from HadGEM2-ES (UK Met Office Hadley Centre) representing a hotter situation (Suppl. Table S1); full details of the models are as provided previously^51^. DIVA climate layers are publicly available as zip files in Dropbox accessible as follows: Ensemble mean, 2050s: https://www.dropbox.com/s/f83edr964ev60ou/all-2050.zip?dl=0; GISS-E2-R: https://www.dropbox.com/s/ml8wyo4hxc64htd/gis2.zip?dl=0; HaddGEM2-ES: https://www.dropbox.com/s/4lctvmwgq7qtu5a/hadg.zip?dl=0.

To evaluate resulting shifts in annual or monthly T_max_ or T_min_, or in mean annual precipitation, we performed point by point calculations by subtracting the corresponding cell values in the grid menu (Diva-GIS 7.5) (Suppl. Fig. S3 (annual), Fig. S4 (monthly)). As described in Jones and Thornton^51,52^, monthly means for rainfall and maximum and minimum air temperature were calculated as anomalies from the historical mean, and then bias-corrected to WorldClim v 2.0^55,56^. The GCM data provide annual deviations for the years through to the end of the current century. Fourth-order polynomials were fitted for each variable to every pixel through time^51^. The GCM models were derived using the delta method (a simple BC) to Coupled Model Intercomparison Project Phase 5 (CMIP5) models and representative concentrations pathways (RCPs) as described in Navarro-Racines et al.^57^. The BC method is effective and robust in generating correct mean data for 30-year climate conditions and widely accepted to minimise the structural bias in the GCM results^57,58^.

To determine the significance of changing climatic parameters, the standard deviation (SD) for the temperature and precipitation were calculated (Suppl. Figs. S5, S6). The standard deviation among mean temperature shifts and precipitation shifts predictions with GISS-2E-R, 17 GCMs and HadGEM2-ES models for Tigray, Amhara, Oromia and SNNP areas was calculated (Suppl. Fig. S4). The SD among mean temperature shifts and precipitation shifts presented based on assumption that the precipitation/temperature data follow a Normal distribution and that the sample SD is a representative SD of the population, the precipitation/temperature data that is outside of the 2*SD is predicted to be significant with a 95% probability (Suppl. Fig. S6). The z-score, which normalizes precipitation shifts in 2050 from the current precipitation values within the four regions, was calculated as:

$$Z\;score = \frac{x - \mu }{\delta }$$

where μ is mean of the sample calculated for precipitation shift in each four regions, x—individual data of precipitation shifts for each point at 5 min resolution, and δ is the standard deviation of the sample.

### Evaluation of model performance and future projections

To illustrate uncertainties of the three GCMs, we constructed maps of the SD between suitability predictions generated with GISS-E2-R, HadGEM2-ES and 17 GCMs GCMs; and a map showing the harmonic mean of the reciprocals of the values in the grid to demonstrate the direction of the average prediction (Suppl. Fig. S5A). The harmonic mean was calculated in GRASS.

$$Harmonic \;mean_{GCM1, \;GCM2,\;GCM3} = \frac{1}{{\left( {{\raise0.7ex\hbox{${\left( {1/x_{GCM1} + 1/x_{GCM2} + 1/x_{GCM3} } \right)}$} \!\mathord{\left/ {\vphantom {{\left( {1/x_{GCM1} + 1/x_{GCM2} + 1/x_{GCM3} } \right)} 3}}\right.\kern-\nulldelimiterspace} \!\lower0.7ex\hbox{$3$}}} \right)}}$$

### Analysis of climate change effect on Ethiopia by 2050

To investigate trends in climate suitability of the three major Ethiopian forage grass species, we first considered how current climates experienced by the regions of study will change compared to 1970–2000 baselines (Suppl. Fig. S3). Notably, all model runs produced significantly increased mean and maximum annual temperature (Suppl. Fig. S3). Similarly, the data harmonisation of the mean and maximum annual temperature and Standard deviation (SD) analysis for the three future climate models indicated temperature increase with high certainty (Fig. S5). In lowlands, we observed little variation between the projections of the scenarios, while projections for some highland areas varied. Similar patterns were observed for maximum temperature trends (Suppl. Fig. S3, central column), with increases from 31–38 °C to 38–45 °C likely in lowland areas. This agrees with IPCC projections that Ethiopia as a whole will experience increased future temperatures^40^. Precipitation patterns were predicted to be relatively unaltered (Suppl. Fig. S3, righthand column), although reduced precipitation was forecast for parts of Tigray and Oromia under the HadGEM2 projection*,* while highland areas of Oromia and SNNP show increased precipitation under the 17 GCM Ensemble mean*.* Z-score and SD analysis for the three future climate models supported the uncertainty in future estimated for annual precipitation, especially in SNNP and Oromia (Suppl. Fig. S3, right insert and Fig. S5).

To determine possible agronomic impact of these changes, we focused on the July–August period during which grass is planted and establishes in Ethiopia^59^. Importantly, mean July–August temperatures are predicted to rise by up to 4 °C, depending on the GCM (Suppl. Fig. S4). We also predict significant reduction in precipitation in all regions during these months under both GISS-E2-R and HadGEM2-ES, where SNNP, Oromia and Amhara could receive up to 30 mm/month less precipitation during a critical time for grass establishment (Suppl. Fig. S3). Z-score and SD analysis of precipitation under GISS-E2-R and HadGEM2-ES projections vs current precipitation indicated high certainty of this (Suppl. Fig. S6). The Ensemble GCM projection predicts increased precipitation in Amhara and Tigray regions in July–August, emphasising ongoing uncertainty concerning future rainfall regime changes in East Africa^60^, again supported by SD analysis of predicted precipitation shifts (Suppl. Fig. S7). Combining these data, we note that under all projections SNNP and Oromia regions will likely experience 2–4 °C temperature increases with simultaneous reductions their (already low) precipitation during the months most critical for grass establishment^61^. Tigray could also be impacted by significant temperature increases (Figs. S4–S7), but precipitation during grass establishment will remain constant or increase slightly in some areas^59^.

### ECOCROP model

To predict regional suitability, we used ECOCROP, a mechanistic model originally implemented in DIVA-GIS^62^ to estimate the impact of predicted climate on crop production. The ECOCROP model estimates species niches from climatic data, using number of growing parameters, minimum and maximum values for temperature, annual precipitation, rainfall intervals, absolute temperature intervals, length of growing season, crop freezing temperature^62,63^. This model allows to evaluate on a monthly basis if there adequate climatic conditions within a growing season for temperature and precipitation, and calculate the climatic suitability of the resulting interactions between rainfall and temperature, based on the Gower distance statistics between each cell on the map and each point, using the value of 19 climatic variables^62,63^.

### Suitability mapping

Crop suitability modelling involves the evaluation of the model and the usage of the selected parameter set(s) to run the model using a certain (set of) climate scenario(s)^63^. The ECOCROP model was used to determine Napier, Rhodes and Buffel grasses suitability to four regions in Ethiopia. To generate outputs, which are presented as a suitability index expressed as a percentage, four climatic and 19 bioclimatic variables (ANUCLIM scheme) that represent annual trends (mean annual temperature, annual precipitation) and extreme or limiting environmental factors (temperature of the coldest and the warmest month, precipitation of the wet and dry year quarters) derived from the monthly temperature and rainfall values were taken in account^56^.

Growth requirements of the three forage species (Napier, Buffel and Rhodes grass; Table 1) were derived from previous studies^20,21,28,64,65^. To project current (1970–2000) and future (2050) suitability for Buffel, Napier and Rhodes grass, we used the environmental variables described above to define cultivation areas based on their suitability for each species using the ECOCROP model in DIVA-GIS^62^. Soil properties were not taken in account for the modelling, as grasses have wide tolerance of soil conditions (Table 1) and studied areas had suitable soil types (Suppl. Fig. S1B). To model the impact of climate change on forage production in the four regions, we compiled factors determining the growth of Buffel, Napier, and Rhodes grasses, using variables pertaining to length of growing season, temperature and precipitation for each of the three species (Suppl. Table S2). For the length of growing season we used start of growth (G_min_), end of growth (G_max_) and length of the growing period (G_used_). For temperature variables we used absolute temperature that will kill the plant (K_tmp_), the minimum average temperature at which the plant will grow (T_min_), minimum average temperature at which the plant will grow optimally (TOp_mn_), maximum average temperature at which the plant will grow optimally (TOp_mx_), maximum average temperature at which the plant will cease to grow (T_max_). For precipitation variables, we used minimum rainfall (mm) during the growing season (R_min_), optimal minimum rainfall (mm) during the growing season (ROp_min_), optimal maximum rainfall (mm) during the growing season (ROp_max_) and maximum rainfall (mm) during the growing season (R_max_). For our model, the suitability was calculated for each crop on the 12 potential growing seasons, under the assumption that if minimum temperature in one of these months is less than KTMP at one day per month, the crop will die, making the suitability score equal to 0%. Alternatively, if temperature of the month is optimal, the score will be equal to 100%. The overall suitability for temperature was calculated as a score of the consecutive number of months needed to complete the growing season. The overall suitability for precipitation was evaluated for the total growing period (defined by G_min_ and G_max_).

We calculated the suitability prediction as described previously^63^, based on precipitation and on the minimum and maximum temperature parameters, where suitability area was a result of combining surfaces obtained from the maximum and minimum temperatures parameter with the same sets of precipitation parameter.

$${SUIT}_{total}=\left\{\begin{array}{cc}{SUIT}_{{T}_{suit}min*{R}_{suit}}& {SUIT}_{{T}_{suit}min*{R}_{suit}}\ne 0; {SUIT}_{{T}_{suit}max*{R}_{suit}}=0\\ {SUIT}_{{T}_{suit}max*{R}_{suit}}& {SUIT}_{{T}_{suit}min*{R}_{suit}}=0; {SUIT}_{{T}_{suit}max*{R}_{suit}}\ne 0\\ \frac{{({SUIT}_{{T}_{suit}min*{R}_{suit}})}^{2}+{({SUIT}_{{T}_{suit}max*{R}_{suit}})}^{2}}{{SUIT}_{{T}_{suit}min*{R}_{suit}}+{SUIT}_{{T}_{suit}max*{R}_{suit}}}& {SUIT}_{{T}_{suit}min*{R}_{suit}}\ne 0; {SUIT}_{{T}_{suit}max*{R}_{suit}}\ne 0\end{array}\right.$$

The combined suitability prediction, which incorporated both temperature and precipitation, was based on the minimum score (%) of the temperature and precipitation for each growing season. Scores of 1–20 were arbitrarily defined as ‘very marginal’, 21–40 as ‘marginal’, 41–60 as ‘suitable’, 61–80 as ‘very suitable’, and 81–100 as ‘excellent’ for grass production. The generated layers were converted to *.sph files using IDRISI GIS Analysis, followed by area measurements in ArcGIS10.2.

To predict shift in areas of suitability, the areas considered suitable or better for each grass species (i.e. a combined suitability score 40–100%) under current conditions were calculated as percentages of the total land area of each region, and the equivalent area of suitability predicted under each future climate model subtracted. Hence, the greater the score the greater the loss of suitable area while a negative score implies a future gain. To model the impacts of these changes on the ability of each region to meet its future forage requirements, two scenarios were developed (Suppl. Fig. S2) using an exploratory approach based on information derived from Ethiopia’s Livestock Master Plan^8^, the ILRI Forage Adoption Study, the Tropical Forages Tool^29^ and the Central Statistics Agency of Ethiopia^3^. Scenario development followed an approach obtained at Scenarios to Strategy Inc. (http://www.scenarios2strategy.com/docs/planning.html). Briefly, the predicted population growth and increased domestic demand were used to predict the future demand for dairy output in Ethiopia; and associated dry matter production modelled under the current and the three future climate scenarios (described more fully in the “Results”). As our focus was on domestic production impacts on Ethiopian food security, we considered only the possibility of meeting the dairy sector’s feed requirements through indigenous production; hence any shortfall implies the need for greater national expenditure and reliance on feed imports.

### Prospects for meeting future livestock demand for dry matter based on land management and land availability in Ethiopia

Since the geographic range of the harvested area of a crop is not only driven by climate, but also by political and socio-economic drivers^63^, it is essential to estimate how land use change and food demand could affect grass availability. Current livestock number and land available for grass production represents a ‘Business as Usual’ approach suitable as a baseline for future analyses, that may vary in either direction depending on demographic shifts or altered urban or land use planning regimes. The International Livestock Research Institute (ILRI) and other organisations have developed proposals for improved productivity (yield) (by altered land management practices and proposed changes to the breeds of cattle reared in Ethiopia)^66,67^. Hence, we modelled outputs of dry matter (DM) from these land areas under both current or optimised land management, current or optimized fertilizer application, and current or amended numbers of indigenous and improved cattle varieties. When we analysed the contribution to DM feed requirement of all grasses in question per region, areas were measured as per Fig. 3 (left panel), followed by calculation of remaining areas and attributing these areas for other grasses. To evaluate the DM feed contribution, DM produced per individual grass species per estimated area was proportioned to total DM feed cattle requirement per region (%). The Uncertainty analysis of areas suitable for Buffel, Napier and Rhodes grass cultivation, under a 15% and 10% land availability scenario under three climate projection models was performed using Monte Carlo simulation model^68^. Based on the power of analysis 84% we selected distribution function to generate 100 new data points for each input. Next, we generated data with 95% confidence interval to quantify R-factor coefficient, explaining the prediction uncertainties for each region with each scenario. The R-factor (Rf) was calculated at 95% confidence based on the following formula,

$$Rf = SD_{N} \times \mathop \sum \limits_{i = 1}^{N} \frac{{\left( {Uq - Lq} \right)}}{N}$$

where SD is the standard deviation of the observed values, N is the number of observed data and Uq and Lq indicate the value of the upper quartile (97.5%) and the lower quartile (2.5%), respectively (Suppl. Table S3).

## Results

### Rhodes grass and Buffel grass remain the most suitable species for forage grass production in Ethiopia under future climate scenarios

**Figure 2:**
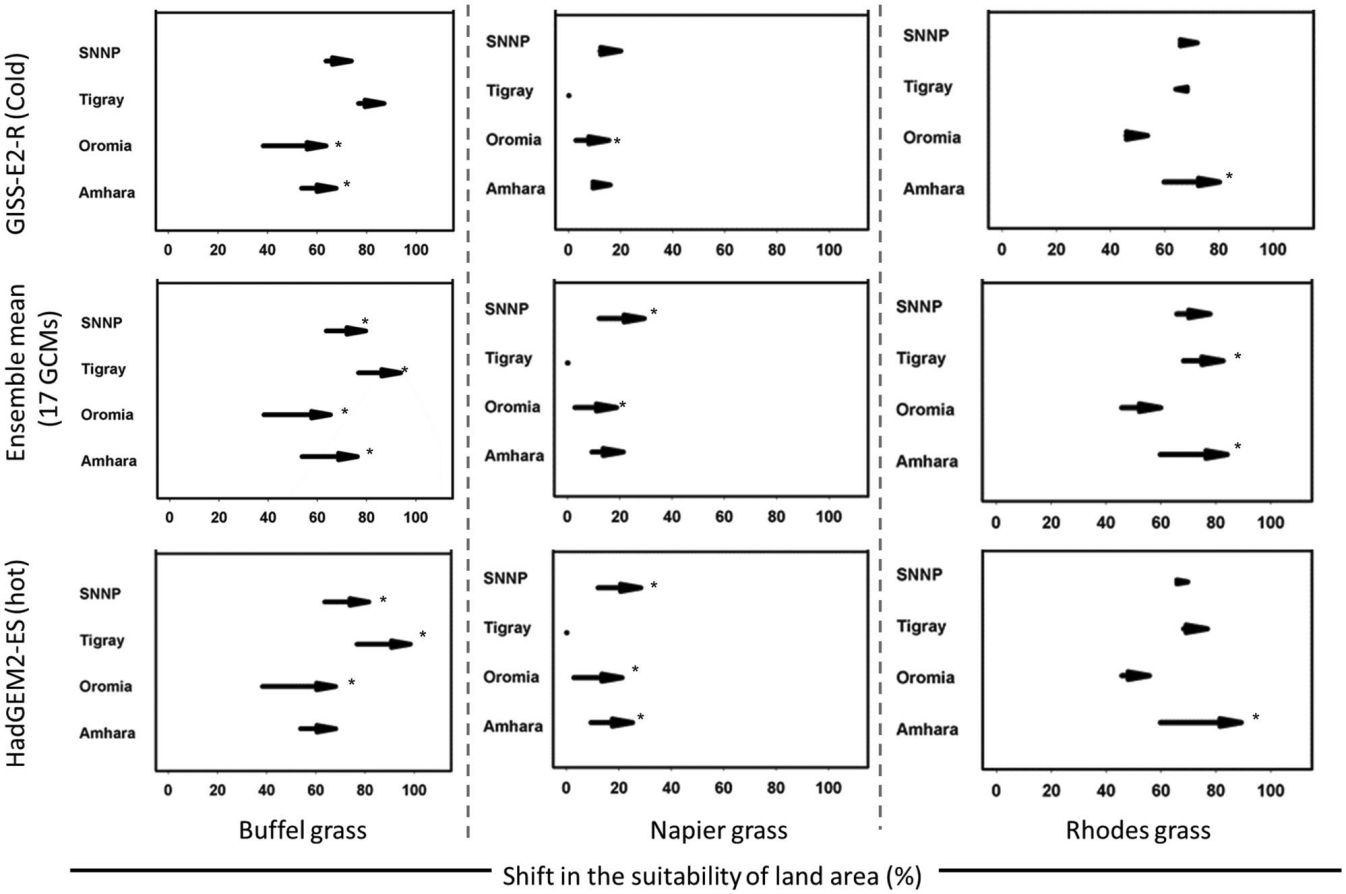
Prediction shifts in areas suitable for the cultivation of Buffel, Napier and Rhodes grasses in regions of Ethiopia. The arrows indicate the shift in land area as a % of the land area of each region from current (1970–2000) conditions to those predicted under different climate change scenarios (left-hand axis), based on predicted impacts on temperature, rainfall and growing season. *Indicates significant change p < 0.05, with Power of analysis = 84.05%.

To determine suitability of different Ethiopia regions (Suppl. Fig. S1) for forage growth under current and future climate scenarios, the mechanical climate suitability ECOCROP model as implemented in DIVA-GIS 7.5 was utilised (see Table S2 for Parameters of Bioclimatic factor variables used for modelling and Methods section for methodology description), followed by area calculations in ArcGIS10.2 and derivation of altered areas of suitability (Fig. 2).

The prediction is based on rainfall and temperature parameters, with minimum values for each growing season used to compute suitability. We set 40% on the 100% scale as our threshold for adequately suitable areas, since climates below this are considered marginal^63^. Buffel and Rhodes grasses are currently suitable to be grown in all regions, with greatest suitability in Tigray: 76.7% and 68.3% of total area respectively (Suppl. Table S4). Throughout, < 15% of land is currently considered suitable for Napier grass, with Tigray having no area, and only 2.9% of Oromia, 9.4% of Amhara and 12.1% of SNNP. To determine climate change impacts on future production, areas of suitability in 2050 were determined, and difference between current and future suitability area calculated. Three climate scenarios did not generate significantly different results (estimated by z-score and ANOVA) for individual grasses suitability area change in specific regions (Fig. 2). Strikingly, under all scenarios, suitable production areas for these three forages will likely increase in three of the four regions. Specifically, in Amhara, Oromia and SNNP, 11–30% more land will become suitable for growing at least one species (Fig. 2, Suppl. Table S4). All area suitability predictions within the study region present inter-model agreements of 80–90%; given the 40% cut-off, SD between the suitability areas was negligible (data not shown).

The best forages to grow in these regions will remain Buffel and Rhodes grasses, as 70–88% of land will be suitable. Tigray will be more exposed to climate risks—although more area will become available for Buffel grass, no new areas will become suitable for Napier grass (Fig. 2). The situation for Rhodes grass is less clear—under one GCM the suitability area falls, while under the others small increases are observed (Fig. 2, Suppl. Table S4). Overall, our analysis confirms that potential land area available for forage cultivation in Ethiopia is likely to increase.

### Land use impacts of meeting future livestock demand for dry matter from forage grasses

To determine how losses of pasture to other land uses will interact with the climate becoming more suitable for grass cultivation, we calculated the total areas which will be available in the four regions when climate parameters are combined with available land conserved at the current 15%^12^, or falling to 10% as predicted by Cellular Automata-Markov (CA-Markov) modelling^49^ (Table 2). We note that these values are likely underestimates as we assume that loss of land to urbanization is equally likely in all areas while in reality urbanization is less likely in high-altitude areas with low suitability for any of the grasses. Notably, in most cases, the area suitable for each grass under the 10% availability model is less than the current availability. In other words, realistic increases in land-use pressure could more than negate the increased area of suitability under a future climate. In some cases, however, the increased suitability of the grasses was such that the total remained higher, e.g. under the colder GCM for Napier and Buffel grasses in Oromia or Rhodes grass in Amhara (Table 2).

**Table 2:** Possible contribution of Buffel, Napier and Rhodes grasses to meet total feed demand (%) under Scenario A and Scenario B, calculated based on the area suitable for individual grass assuming that all land will be used only for monocrop culture production under predicted future conditions, if (A) 15% and (B) 10% of total land is used for pasture. F (LY) = ‘fertilized (business as usual, average yield 5.7 tons per ha)’, F (HY) ‘fertilized (highest reported yield for the species as per Table 1)’.

(A) Possible contribution from each grass species to feed demand (%)							
Scenario	Region	Buffel grass		Napier grass		Rhodes grass	
		F (LY)	F (HY)	F (LY)	F (HY)	F (LY)	F (HY)
Current	Amhara	25	69	4	23	27	87
	Oromia	22	62	2	9	26	83
	Tigray	32	88	0	0	28	89
	SNNP	30	85	6	30	31	99
**Predicted Scenario A if 15% of total land is used for pasture**							
Future GISS-E2-R_Cold	Amhara	29	82	7	36	35	109
	Oromia	32	91	8	41	27	86
	Tigray	32	91	0	0	24	75
	SNNP	29	80	8	41	28	88
Future_17 GCMs	Amhara	33	93	9	48	36	115
	Oromia	33	93	10	50	31	96
	Tigray	35	98	0	0	31	97
	SNNP	31	87	11	60	30	95
Future HadGEM2-ES_hot	Amhara	29	83	11	57	39	122
	Oromia	35	97	11	57	28	90
	Tigray	37	103	0	0	29	90
	SNNP	32	89	11	58	27	85
**Predicted Scenario B if 15% of total land is used for pasture**							
Future GISS-E2-R_Cold	Amhara	19	52	4	23	22	69
	Oromia	21	58	5	26	17	55
	Tigray	20	57	0	0	15	48
	SNNP	18	51	5	26	18	56
Future_17 GCMs	Amhara	21	59	6	31	23	73
	Oromia	21	59	6	32	19	61
	Tigray	22	62	0	0	19	61
	SNNP	20	55	7	38	19	60
Future HadGEM2-ES_hot	Amhara	19	52	7	36	24	77
	Oromia	22	62	7	36	18	57
	Tigray	23	65	0	0	18	57
	SNNP	20	56	7	37	17	54

(B) Possible contribution from each grass species to feed demand (%)							
Scenario	Region	Buffel grass		Napier grass		Rhodes grass	
		F (LY)	F (HY)	F (LY)	F (HY)	F (LY)	F (HY)
Current	Amhara	25	69	4	23	27	87
	Oromia	22	62	2	9	26	83
	Tigray	32	88	0	0	28	89
	SNNP	30	85	6	30	31	99
**Predicted Scenario A if 10% of total land is used for pasture**							
Future GISS-E2-R_Cold	Amhara	19	55	5	24	23	73
	Oromia	22	61	5	28	18	58
	Tigray	22	61	0	0	16	50
	SNNP	19	54	5	27	19	59
Future_17 GCMs	Amhara	22	62	6	32	24	77
	Oromia	22	62	6	34	20	64
	Tigray	23	65	0	0	20	65
	SNNP	21	58	8	40	20	64
Future HadGEM2-ES_hot	Amhara	20	55	7	38	26	81
	Oromia	23	65	7	38	19	60
	Tigray	24	68	0	0	19	60
	SNNP	21	59	7	39	18	57
**Predicted scenario B if 10% of total land is used for pasture**							
Future GISS-E2-R_Cold	Amhara	12	35	3	15	15	46
	Oromia	14	38	3	18	12	36
	Tigray	14	38	0	0	10	32
	SNNP	12	34	3	17	12	37
Future_17 GCMs	Amhara	14	39	4	20	15	48
	Oromia	14	39	4	21	13	41
	Tigray	15	41	0	0	13	41
	SNNP	13	37	5	25	13	40
Future HadGEM2-ES_hot	Amhara	12	35	5	24	16	51
	Oromia	15	41	5	24	12	38
	Tigray	15	43	0	0	12	38
	SNNP	13	37	5	24	11	36

All values are presented as ± 28%, based on the mean, min and max yield for each grass species under Scenario A and Scenario B.

Among livestock, cattle have the highest energy requirements and drive 82–86% of annual feed requirement per region^7^. To determine the DM currently required by the total dairy herd within the region, and how future changes might interact with climate change impacts, we again considered the two scenarios: “Scenario A” and “Scenario B” (Suppl. Fig. S2).

### Increased DM demand for livestock can only be met under future climate change scenarios if forage grassland management is improved

To evaluate the contribution to cattle feed requirements, calculations were made assuming 2.5% DM feed per kg hd^−1^ d^−1^ for local and exotic cattle breeds of weight ~ 200 and ~ 600 kg, respectively^4^, and assuming that total numbers of cattle remain constant (Table 3). The number of hectares sufficient to provide the total dry matter (DM) required per region was calculated using the yield per hectare under different land management practices, in particular fertiliser application (Table 1; Suppl. Table S4). We found that intensification of the dairy herd will indeed increase total DM required: substitution of 40% of head with exotic breeds would cause increased DM demand of 158%, even assuming no increase in herd size (Table 3).

**Table 3:** Total dairy livestock numbers and resulting feed requirements under Scenario A (93% local stock and 7% exotic breeds) and Scenario B (60% local stock and 40% exotic breeds).

Region	Total number of dairy cattle^3^	Total DM currently required for livestock, tons^7^	Current grass feed deficit (% of grass DM produced to total DM required for livestock)	Predicted total dry matter required for cattle (metric tons)	
				Scenario A	Scenario B
Amhara	14,710,911	34,270,000	− 59.4	30,606,050	48,325,343
Oromia	22,925,730	50,160,000	− 57.7	47,696,981	75,311,023
Tigray	4,578,181	10,260,000	− 53.8	9,524,906	15,039,325
SNNP	11,215,636	22,540,000	− 69.3	2,3334,131	36,843,364
Total	10,595,380	117,230,000	− 240%	111,162,068	175,519,055

To determine in more detail the impacts of increased forage demand over the different Ethiopian regions, we compared feed demand to DM output for the two scenarios in each region under the different climate models with both land-availability scenarios. For each forage, productivity was estimated for lands under both “poor” and “optimal” management systems (Table 1). Specifically, we considered the percentage of available agricultural land w needed to meet regional supply with current low yields (~ 5.7 tons ha^−1^, based on grass DM production, per region, per 15% land used for pasture), denoted F(LY); and assuming best possible yields under appropriate fertiliser administration, denoted F(HY)^7,28,64^. For simplicity, we considered each of the regions in isolation, discounting any possible inter-regional trade. The contribution of each species to total feed demand was calculated assuming that 15% of land remains available for pasture (Table 2A), as well as the more severe scenario in which it falls to 10% (Table 2B). In both cases, we modelled scenario A (current herd composition and a slight increase of exotic breads from 3 to 7% of the total^3^), and scenario B (herd composition incorporating 40% high-yielding elite cattle) with the three sets of GCM outputs for 2050. Strikingly, feed demands of the current herd compositions^7^, cannot be met current management practices (Table 3). Moreover, none of the three grass species is able to satisfy current feed demand under F(LY) conditions (Table 2A). Only with the best management practice could Rhodes grass contribute 83–99% of current feed demand. Napier grass is unable to meet existing feed demand, even with the best agronomic practices, only accounting for at most 30% of demand (Table 2A).

Next, we considered whether feed demand could be met in 2050, taking into account increased suitability area for three modelled grass species predicted by three projected GCM scenarios (Suppl. Table S1). Modelling “Scenario A” and “B” based on low management practice, where yield remains the same as current (5.7 tons DM per Ha), revealed that the future feed demand could *not* be met even with 15% of pasture land remaining available (Table 2A,B). However, with improved grass yield, nearly all cattle feed demand could be met by growing Rhodes or Napier grasses in the suitable areas (41–100% suitability score; see Methods, Table S3). Importantly, under future climate projection, Rhodes grass, yielding 18 tons/ha, could be harvested in excess in Amhara (+ 9–22%) (Table 2A, F(HY)). Napier grass, despite having the highest productivity and increased suitability areas, could only satisfy at most 50% of feed demand under Scenario A.

Unsurprisingly, a more intensified herd composition (Scenario B, 40% exotic breeds) significantly increased DM cattle feed demand (Table 3). Notably, under 15% land availability with Scenario B, regardless of which climate model is considered, no region could meet its predicted DM requirement if grass productivity is accessed individually (Table 2A). At most, Rhodes grass could contribute 54–77% of total cattle feed demand in 2050 (Table 2A, Scenario B).

**Figure 3:**
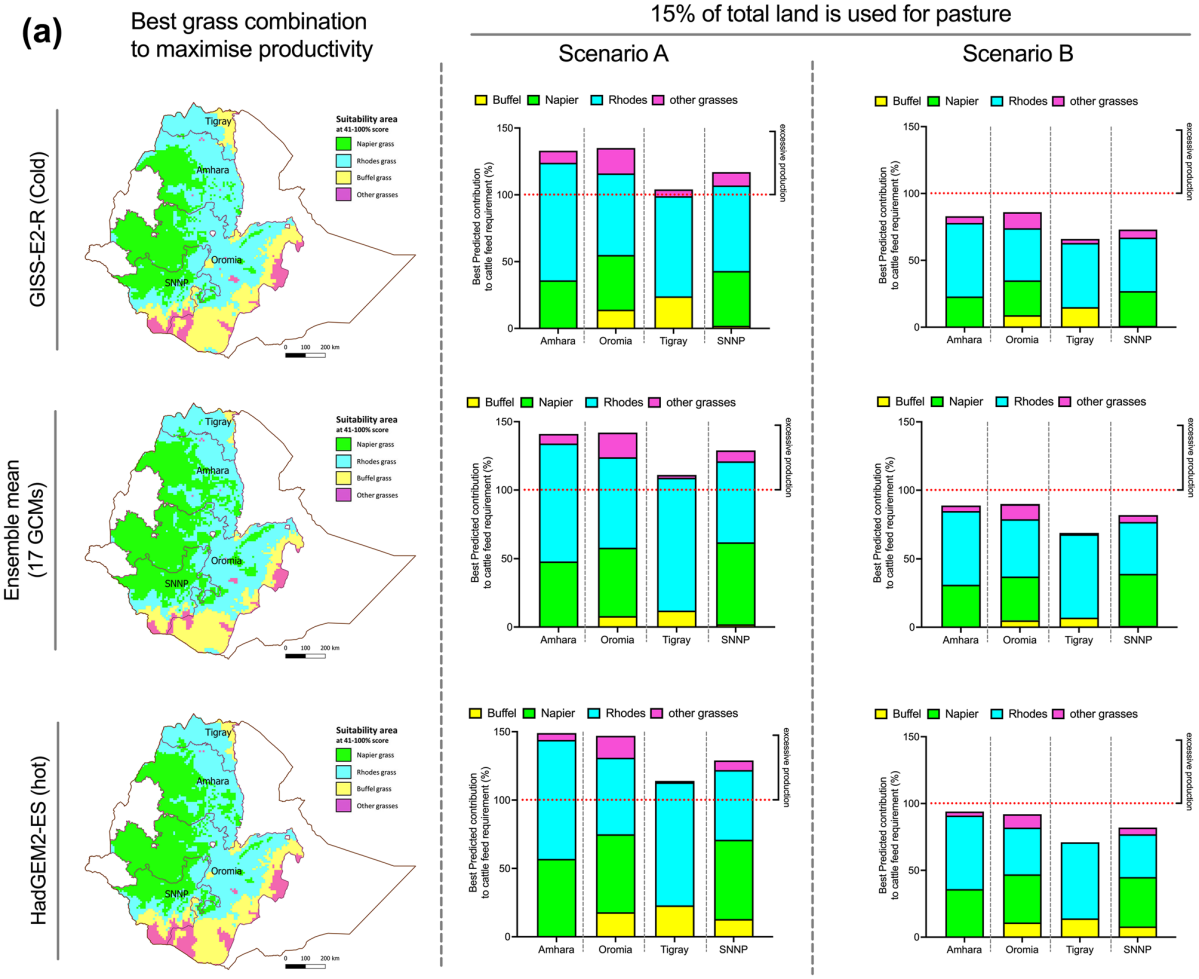

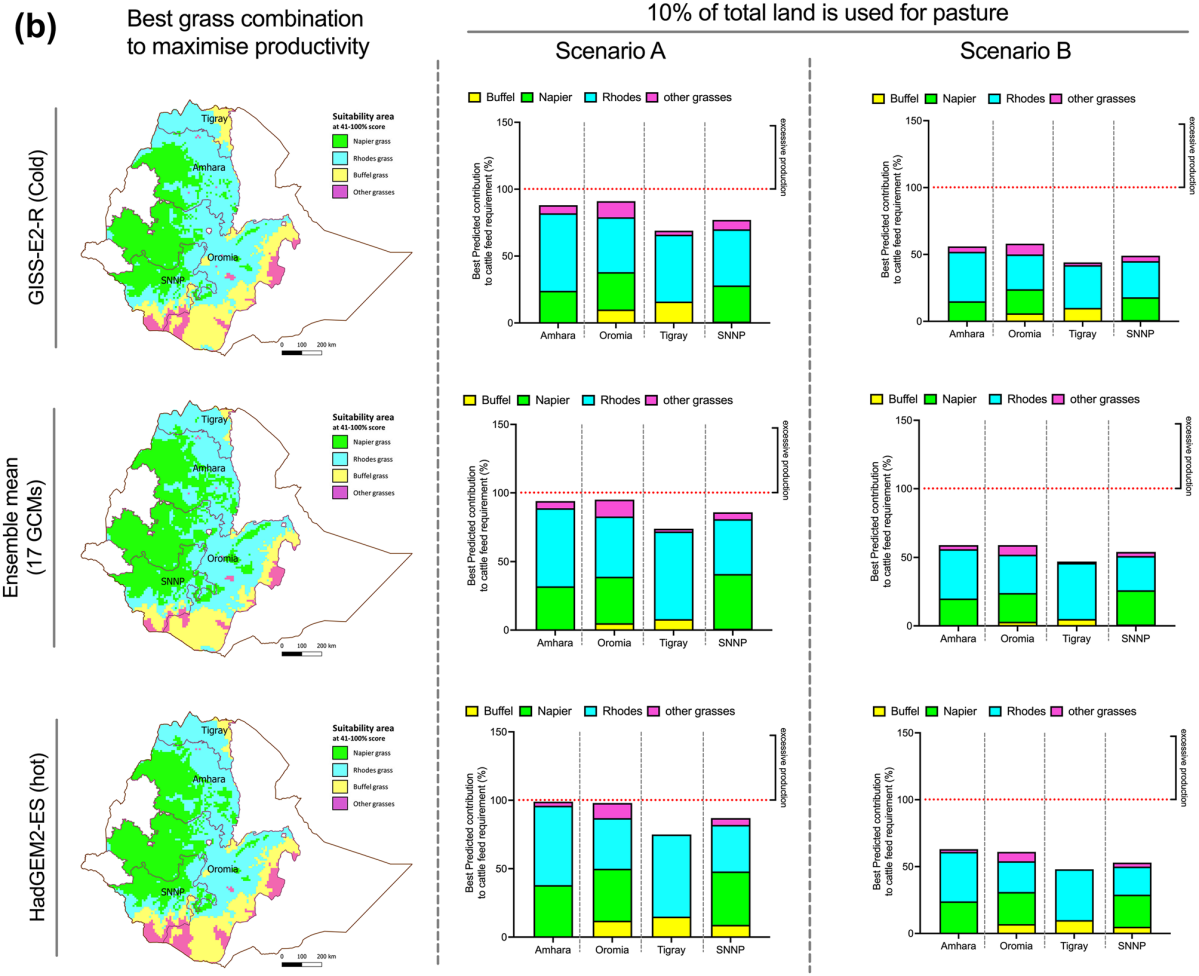
(**A**) Possible contribution of Buffel, Napier and Rhodes grasses to meet total cattle feed demand (%) under Scenario A and Scenario B, if 15% of total land is used for pasture. The feed production is calculated based on assumption that land will be allocated to crops according to suitability × productivity under predicted future conditions, based on F (HY) ‘fertilized (highest reported yield for the species as per Table 1)’. ‘Other grasses’ indicates any other grass species under cultivation in areas not suitable for Buffel, Rhodes or Napier grasses. Yield for other grass species is averaged to 5.7 tons per ha. Maps were generated using software ArcGIS Pro software (https://pro.arcgis.com). (**B**) Contribution of Buffel, Napier and Rhodes grasses to meet total cattle feed demand (%) under Scenario A and Scenario B, if 10% of land is used for pasture. Feed production assumes that land will be allocated to crops according to suitability × productivity under predicted future conditions, based on F (HY) ‘fertilized (highest reported yield for the species as per Table 1)’. ‘Other grasses’ indicates any other grass species under cultivation in areas not suitable for Buffel, Rhodes or Napier grasses. Yield for other grass species is averaged to 5.7 tons ha^−1^. Maps were generated using software ArcGIS Pro software (https://pro.arcgis.com).

Napier grass has the highest productivity among the three grasses, but a smaller suitable growing area, while Rhodes and Buffel grasses have lower yields but can be cultivated on most areas in all four regions. Hence, we developed grass cultivation models, wherein land is allocated to the crop based on best productivity and best suitability under the predicted future conditions for that area. Combined contributions of Rhodes, Buffel and Napier grasses under best productivity models were evaluated under both “Scenario A” and “B” and two land-access models (Fig. 3A,B). The areas unsuitable for Rhodes, Napier or Buffel grass cultivation were attributed to other grass species, assuming yields of 5.7 tons/ha^7^.

Under appropriate soil management systems, if 15% of land is used for pasture and land is allocated to grass species based on combined suitability and productivity models, all regions will be able to meet cattle feed demand if herd composition follows Scenario A (Fig. 3), by taking advantage of the increased total area suitable for these grasses projected previously (Fig. 2). Moreover, feed will be generated in excesses of 5–10% in Tigray and 30–50% in Amhara, Oromia and SNNP. Importantly, under Scenario A, if total available land for pasture falls to 10%, only severe climate change (HadGEM2-ES projection), could ensure feed security in Amhara and Oromia, while under the other projections cattle feed will be in deficit (Fig. 3B, Suppl. Table S3).

However, to meet food security for a dairy herd with 40% exotic cattle (Scenario B), with 15% available land for pasture, no regions will generate enough DM to meet demand; this is exacerbated if cultivated land falls to 10% of total area (Fig. 3B). However, if poor management systems are assumed, then the amount of forage produced falls short of what is required in both Scenarios A and B, potentially causing greater feed and food insecurity and this perdition has high level of certainty (Suppl. Table S3).

We conclude that, if pasture and forage land fall to 10% of total land area by 2050 (as projected from current patterns^11^), significant improvement of agricultural practices, genetic gain for forage germplasm performance and/or consideration of alternative forage species will be needed to produce sufficient livestock feed for the growing population of these regions of Ethiopia, even assuming that no increase in the size of the herd occurs over this period.

## Discussion

Climate change adaptation of cropping systems will require careful consideration of the overall impacts on crop suitability, especially in areas considered vulnerable to food insecurity. In this study, we used ECOCROP modelling to project combined impacts of climate change on areas of Ethiopia suitable for growing three major forage grasses supporting livestock production. We predict that in three of the four regions of Ethiopia studied, the land suitability for Buffel, Napier and Rhodes grasses is likely to significantly increase, indicating that climate change is unlikely to be a threat to their cultivation, at least not when considered in isolation from other land-use pressures. However, differences between different levels of global heating can alter these effects, and potentially complex interactions with other ongoing trends, both global and regional^69,70^, will need careful consideration for reliable policy recommendations.

### Growth and productivity of Ethiopian forage grasses under different conditions

In this study we have established a baseline for likely changes in growing area suitability for three forage grasses on the basis of predicted changes in temperature and precipitation patterns, and through impacts on the growing season. We have furthermore combined this model with likely scenarios of land-use availability and dairy herd composition. Models of this kind are inherently limited, as they are based on bioclimatic factors, without considering soil requirements, pests, or diseases, nor altered CO_2_ level. Such factors could all increase or decrease possible output of the species considered. For example, Buffel grass displays increased biomass, plant height, leaf length, leaf width and general improved performance under elevated CO_2_^71^. Similarly, Rhodes grass shows higher plant height and greater DM under very high (650 ppm) CO_2_^72^. Hence, climate change and ongoing atmospheric CO_2_ enrichment could potentially create opportunities for increased productivity of forages in many parts of Ethiopia, even in the case of C4 species which benefit less from such effects^38^. These results could alleviate pressures on land use observed in our results, and agree with counter-intuitive findings from multi-year studies of grasses in permanent pasture^73^, yet could themselves be confounded by changes to water availability. It has been demonstrated that long-term adaptations of tree species, soybeans and other plants to CO_2_ enrichment are to reduce stomatal opening^38,74^. Hence one possible outcome is that plants adapt by maintaining biomass production while increasing water use efficiency without any gain in yield^75^ although interactions with nutrient availability and other factors may further complicate this^76,77^.

Our modelling approach has certain limitations which should be addressed in future work: we omitted carbon intensity as life-cycle analysis was not performed; we also used only high GHG emissions scenario, to assess the strongest impact on the livestock production system in Ethiopia; we have not for instance taken account of waterlogging—which has complex dynamic interactions on the soil–plant–climate continuum—or the impact of other extreme weather events. However, climate prediction using 17GCMS and HadGEM2-ES models indicate increased precipitation in Tigray and Amhara regions which could have possible negative impacts on the land suitability for Buffel grass production due to waterlogging. To address this would require collection of direct experimental data on the response of the three grasses to the waterlogging stress and its impacts on biomass and yield, followed by applying suitable algorithms to mimic waterlogging (e.g. SDI or CROPR^78^). In our models, we took an assumption that “average” conditions occur regularly, and our analysis does not cover the possibility that future climate “averages” are a collection of oscillating extreme values, what could significantly impact overall crop production system.

In some cases, monthly variability will be key to the outcome of a forage in an area. Our modelling revealed for example that the Tigray region will become less suitable for growth of Buffel grass, based on the minimum temperature, precipitation score and rainfall during the growing season. Severe alteration of precipitation and temperature regimes during important agricultural months (Fig. 3) are the main reason for this reduced suitability. It may therefore be necessary to source or breed Buffel grass cultivars with improved drought tolerance, or promote use of alternative species in these areas. Importantly, no significant difference for Rhodes grass was predicted for Tigray, despite its smaller area of suitability overall, presumably because this grass is more drought-tolerant. Indeed, its deep roots, reaching down to 4.7 m^21^, allow Rhodes grass to withstand long dry periods^29^.

A principal limitation of the area-based model which we employed is that general effects (yield, water use change) are not necessarily reflected in area of suitability (Suppl. Table S4). This means that total yield could alter in ways which have not been modelled. Genetic selection of germplasm adapted for the conditions that become widespread under future scenarios (or introgression of favourable genetic diversity) could allow yields to be enhanced further and assist sustainable intensification^79^. This will however rely upon accurate identification of areas which will remain at minimal risk of lethal levels of either heat or drought.

### Impact on trends in the Ethiopian dairy sector

Given the total number of dairy cows in each region from the Ethiopian CSA’s census^3^, the continued productivity of forage grasses provides the potential to meet the dry matter needs of Ethiopia’s livestock sector under future climates, but only given certain conditions and trade-offs (Figs. 2 and 3). At present, a shortage of animal feed is considered the main limitation on the productivity of Ethiopian livestock systems, along with unsuitable land-use patterns^8,9^. There is also a lack of investment in land improvement and management when compared with other agricultural sectors^8^. The reasons for animal feed shortages are diverse, and include low adoption of forage grasses, influenced by lack of economically active labour, lack of participation or membership of farmers in farming organizations; and the extent and nature of extension advice^80^. Fulfilling the growing potential of forage use will require these bottlenecks to be addressed, while recognising and addressing the barriers to uptake of new techniques^81^. This analysis would also need to incorporate some consideration of other nutritional qualities, including energy and crude protein. This will be particularly critical when considering the ability to maintain herds with increased numbers of improved exotic cattle which will have specific feeding requirements.

Research into these nutritional qualities would also be needed to assess how and to what extent they are likely to change under altered climates, as metabolized energy and crude protein are essential factors for positive feed balance. Indeed, on a country level current dry matter deficiency is 9%, while crude protein and metabolized energy deficiency is 42% and 45% respectively^7^ The biochemical composition of the feed prior to its inclusion into livestock feeding regime will also need to be assessed^82^, taking into account the differences in nutritional requirements between local zebu cattle and exotic introduced breeds^83^. It would also need to take account of the predicted loss of digestibility of many forage species under future climate stress conditions^84–86^, as shown in other crops^87,88^. Such conditions may prevail even in areas considered ‘suitable’ in our analysis, although timeframe will need careful consideration also^89^. There is currently significant pressure to increase the proportion of more productive exotic breeds (Fig. S2): for example, local breeds have a lactation period lasting 50–74 days and a mean milk yield per day of 1.9–2.1 l, resulting in 90–150 l of milk per cow per lactation period. Exotic breeds, whether two-way or three-way breed crosses, typically have lactation periods of 366–411 days and can produce 5.8 l per day, making exotic breeds at least 3.5 times more efficient per unit feed consumed compared to local breeds^3^. However, it should also be considered that exotic breeds require 3.8-fold more dry matter per day when compared to local breeds. Meeting this demand may be possible, but again only if appropriate management is practiced and fertilizer made widely available (Table 2). Even if the forage yield gap is closed, the proportions of the total agricultural land area in the four regions studied will still need to be increased to meet this demand (Fig. 3A vs Fig. 3B). Hence greater focus on breeding for improved forage yield will be increasingly important to relieve pressure on other land uses^14^.

### Potential interaction with land-use patterns in Ethiopia’s regions

Any future increases in Ethiopia’s forage production needs to be balanced against other land uses, including cultivation of staple crops such as maize, teff, and wheat, production of plantation crops as part of ongoing plans for export expansion, and the needs of the dairy herd balanced against production of beef and other livestock^9^. Contribution to preserved areas and regions vital for integrity of ecosystem services would also need to be maintained, including commitments to afforestation and land rehabilitation as set out in Ethiopia’s Nationally Determined Contribution (NDC) to the UNFCCC’s Paris Agreement^90^.

This could lead to livestock being fed with crop residues, which are generally of poorer quality as feed resources when fed without any nutrient supplements to livestock, with high levels of fibre, low to moderate digestibility and low levels of nitrogen and minerals. Indeed, whether land will be used for crops, forage grasses or other purposes will depend upon the resulting income. Major losses of crop yields due to drought as a result of climate change can be anticipated, particularly for maize^91^, while forages such as Buffel and Rhodes grass will continue to be suitable for cultivation. This may influence farmers to consider integrating forage production into their cropping enterprises as means to supplement profits. Hence, forages could form an important component of Ethiopian smallholders’ biophysical and economic adaptation strategies to climate change. As Napier grass is more drought-sensitive, it may instead be less suitable, even if it can produce higher yields when sufficient water is available.

Farmers could also consider using marginal lands (e.g. saline soils), rather than using cropping land for forage production (Buffel grass can be grown on saline soil). Ethiopia has *c.* 11 million hectares of such land^11^ although these are mostly in regions where dairy livestock farming is not practised, leading to higher transportation costs. The use of marginal lands for feed production would however be beneficial not only for livestock but also as a contribution to increased land-use efficiency^11^. Finally, consideration should be given to whether farmers should apply conservation agriculture approaches by intercropping forage legumes with forage grasses in order to boost soil nitrogen content^92,93^. Forage and browse legume supplementation has also been found to be effective in improving the utilization of crop residues^94,95^. However, modelling of climate impacts on areas suitable for legume growth in the future will be needed to ensure the development of optimised mixed cropping systems.

As feed shortages are a key limitation on productivity within Ethiopian livestock systems, consideration should be given to how best to take advantage of potential increases in forage grass output between now and 2050, and what trade-offs with other land use options and environmental impacts will be required. Given that the forage species and regions account for the majority of dairy production in Ethiopia, the findings of this study are likely to be more generally applicable not only across Ethiopia, but also across neighbouring countries.

## Conclusions

The results of this study indicate that climate change will impact on future forage production in Ethiopia, with Buffel grass production in the Tigray region being adversely affected, but with overall positive effects for Rhodes and Napier grass, and for Buffel grass in three regions (Amhara, Oromia and the SNNP). However, to meet future demands of the Ethiopian dairy cattle sector the careful consideration of forage grass breeding targets, selection of germplasm that will have maximum productivity under changing climate conditions, as well as effective grass and livestock management strategies are essential.

## Supplementary Information

Supplementary Information.
