# Supplementary material for: Climate change and land-use change impacts on future availability of forage grass species for Ethiopian dairy systems
Source: Sci Rep. 2022 Nov 28;12:20512. doi: 10.1038/s41598-022-23461-w (PMC9705545; doi:10.1038/s41598-022-23461-w)
Supplement: Supplementary file 1 — Supplementary Information. [file 41598_2022_23461_MOESM1_ESM.docx]

**Supplementary Information**

**Climate change and land-use change impacts on future availability of forage grass species for Ethiopian dairy systems**

Galina Brychkova, Kelebogile Kekae, Peter C. McKeown, Jean Hanson, Chris S. Jones, Philip Thornton, Charles Spillane

**SUPPLEMENTARY FIGURES**

Supplementary Figure S 1. Description of the study area. A. Africa continent and location of the four areas of study (Amhara, Oromia, Tigray and Southern Nations, Nationalities, and People’s Region (SNNP)) in Ethiopia. Soil texture types (B) and hydrological zones (C) for Tigray, Amhara, Oromia and SNNP areas (adapted from ^80^). D. Köppen-Geiger climate classification at 1-km resolution scale for the current conditions (1980–2016), left panel, and right panel shows the future map (2071–2100) derived from revised global maps using published color scheme ^3^. The classification is based on criteria like temperature, which allows for different local vegetation growth. The present-day map is constructed from an ensemble of four high-resolution, topographically-corrected climatic maps; the future map is derived from an ensemble of 32 climate model projections (scenario RCP8.5), by superimposing the projected climate change anomaly on the baseline high-resolution climatic maps. Af-Tropical rainforest, Am – Tropical Monsoon, Aw – Tropical Savannah; BWh Arid desert hot, BWk-Arid desert cold, BSh-Arid Steppe hot, BSk-Arid Steppe cold; Csa – Temperate dry hot summer , Csb - Temperate dry warm summer, Csc - Temperate dry cold summer, Cwa - Temperate dry winter hot summer, Cwb - Temperate dry winter warm summer, Cwc - Temperate dry winter cold summer, Cfa - Temperate without dry season hot summer, Cfb - Temperate without dry season warm summer, Cfc - Temperate without dry season cold summer. 3

Supplementary Figure S 2. Scenario development for interpreting the influence of increasing human population and its implications on livestock products demands; and demands for feed resources by the livestock population needed to increase productivity accordingly. 4

Supplementary Figure S 3. Changes to the Ethiopian climate by 2050 as predicted by three future climate models, when compared to a 1970-2000 baseline: mean and max annual temperature, annual precipitation and Z-score of annual precipitation shift for each model. Changes shown are for the regions of Tigray, Amhara, Oromia and the SNNP Region. 5

Supplementary Figure S 4. Shifts in mean temperature and precipitation during July and August for Tigray, Oromia, Amhara and SNNP predicted by three future climate projections (2050) compared to 1970-2000 baselines. 6

Supplementary Figure S 5.Uncertainties in GCMs climate prediction across the region. Harmonic mean of annual mean and maximum temperature and precipitation (A) and Standard deviation among predictions (B) for Tigray, Amhara, Oromia and SNNP areas. 7

Supplementary Figure S 6. Evaluation of uncertainties in precipitation change in 2050. Standard deviation among precipitation predictions with GISS-2E-R, 17 GCMs and HadGEM2-ES models vs current climate data across for Tigray, Amhara, Oromia and SNNP areas. Z-score of precipitation change in the four regions in 2050. 8

Supplementary Figure S 7. Standard deviation among mean temperature shifts and precipitation shifts predictions with GISS-2E-R, 17 GCMs and HadGEM2-ES models for Tigray, Amhara, Oromia and SNNP areas. 9

*
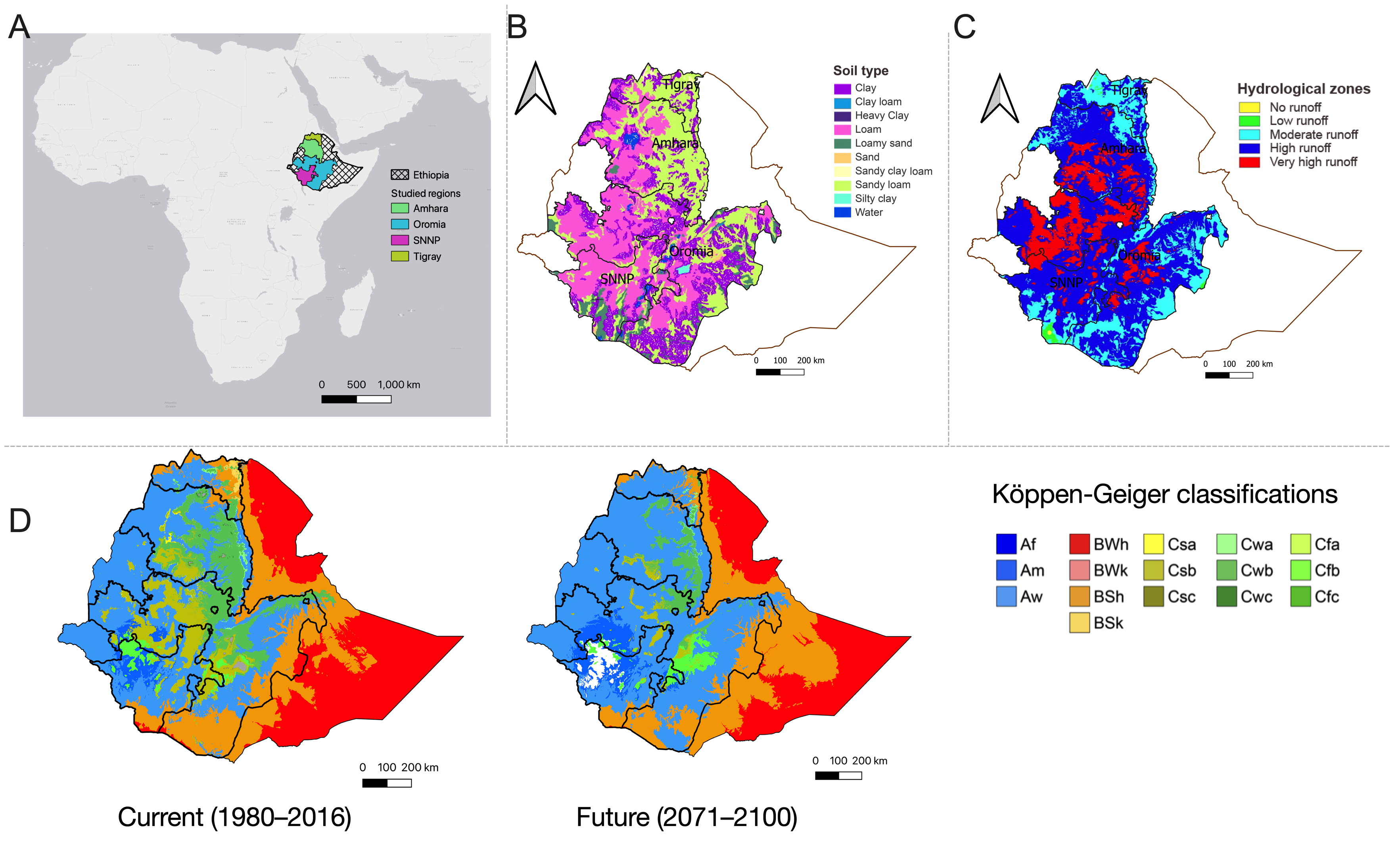
*

Supplementary Figure S 1. Description of the study area. (A). Africa continent and location of the four areas of study (Amhara, Oromia, Tigray and Southern Nations, Nationalities, and People’s Region (SNNP)) in Ethiopia. Soil texture types (B) and hydrological zones (C) for Tigray, Amhara, Oromia and SNNP areas (adapted from^1^). (D). Köppen-Geiger climate classification at 1-km resolution scale for the current conditions (1980–2016), left panel, and right panel shows the future map (2071–2100) derived from revised global maps using published color scheme^2,3^. The classification is based on criteria like temperature, which allows for different local vegetation growth. The present-day map is constructed from an ensemble of four high-resolution, topographically-corrected climatic maps; the future map is derived from an ensemble of 32 climate model projections (scenario RCP8.5), by superimposing the projected climate change anomaly on the baseline high-resolution climatic maps. Af-Tropical rainforest, Am – Tropical Monsoon, Aw – Tropical Savannah; BWh Arid desert hot, BWk-Arid desert cold, BSh-Arid Steppe hot, BSk-Arid Steppe cold; Csa – Temperate dry hot summer , Csb - Temperate dry warm summer, Csc - Temperate dry cold summer, Cwa - Temperate dry winter hot summer, Cwb - Temperate dry winter warm summer, Cwc - Temperate dry winter cold summer, Cfa - Temperate without dry season hot summer, Cfb - Temperate without dry season warm summer, Cfc - Temperate without dry season cold summer. Maps were generated using software ArcGIS Pro software (https://pro.arcgis.com).


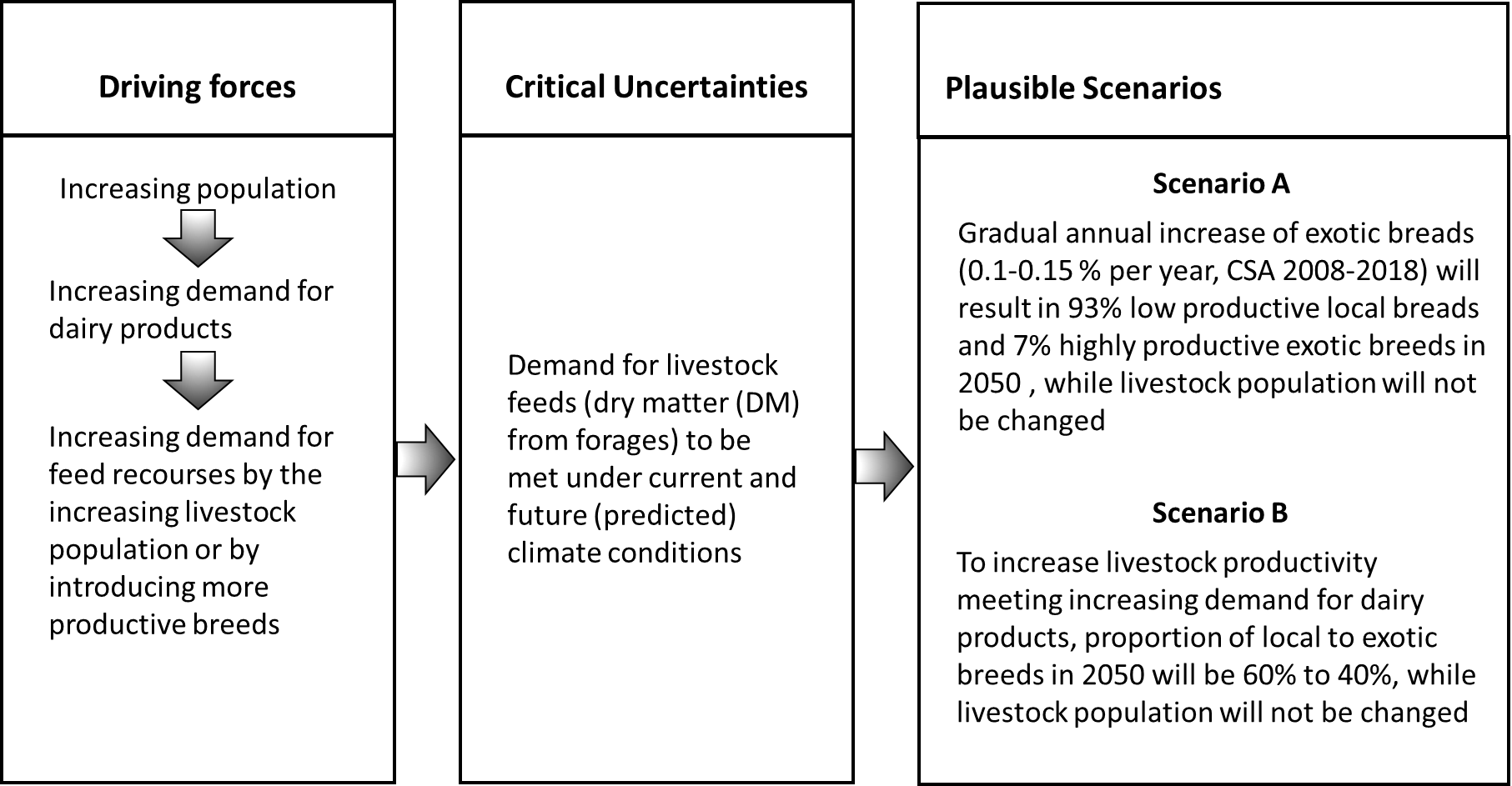


Supplementary Figure S 2. Scenario development for interpreting the influence of increasing human population and its implications on livestock products demands; and demands for feed resources by the livestock population needed to increase productivity accordingly.

**
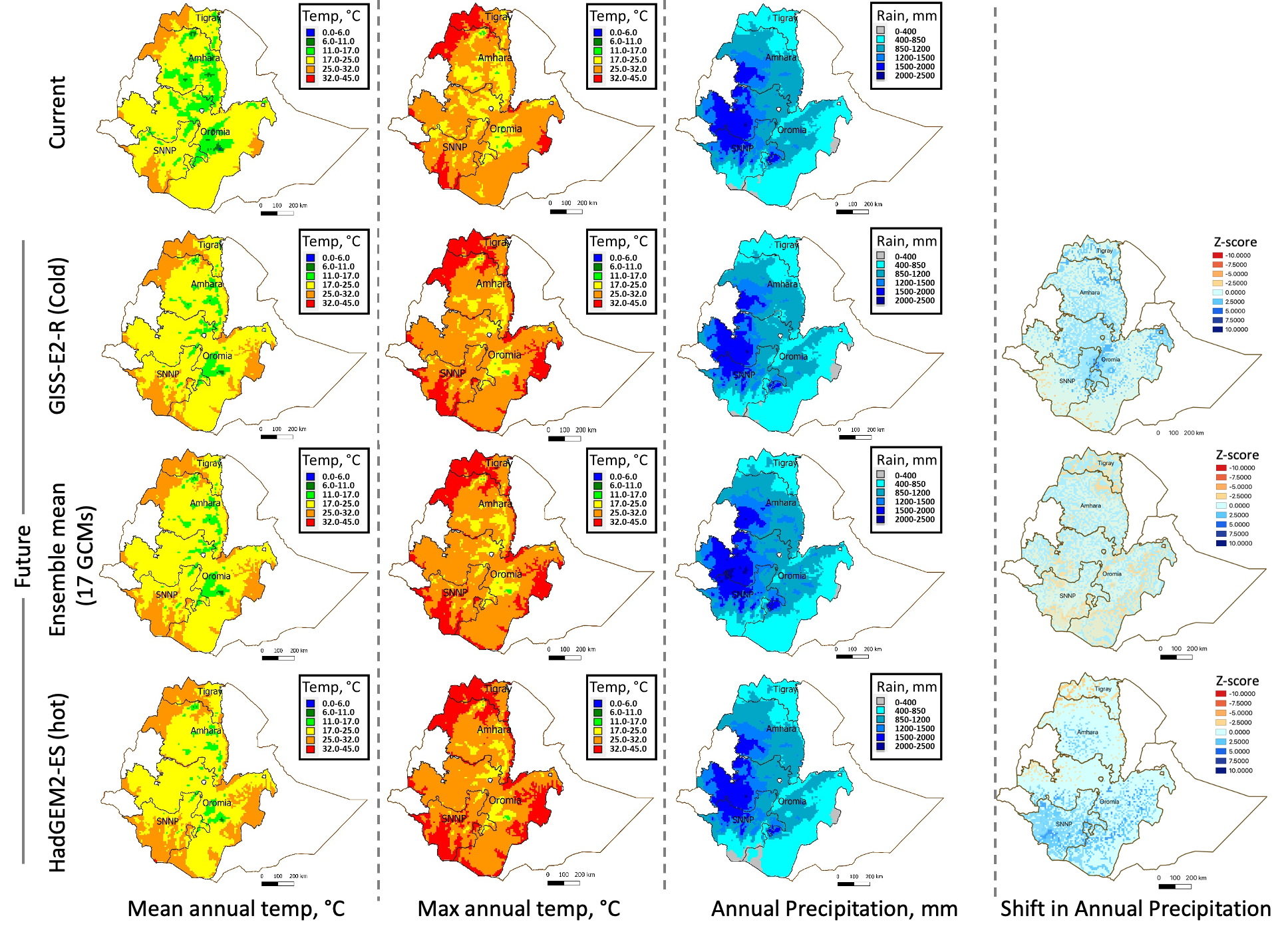
**

Supplementary Figure S 3. Changes to the Ethiopian climate by 2050 as predicted by three future climate models, when compared to a 1970-2000 baseline: mean and max annual temperature, annual precipitation and Z-score of annual precipitation shift for each model. Changes shown are for the regions of Tigray, Amhara, Oromia and the SNNP Region. Maps were generated using software ArcGIS Pro software (https://pro.arcgis.com).


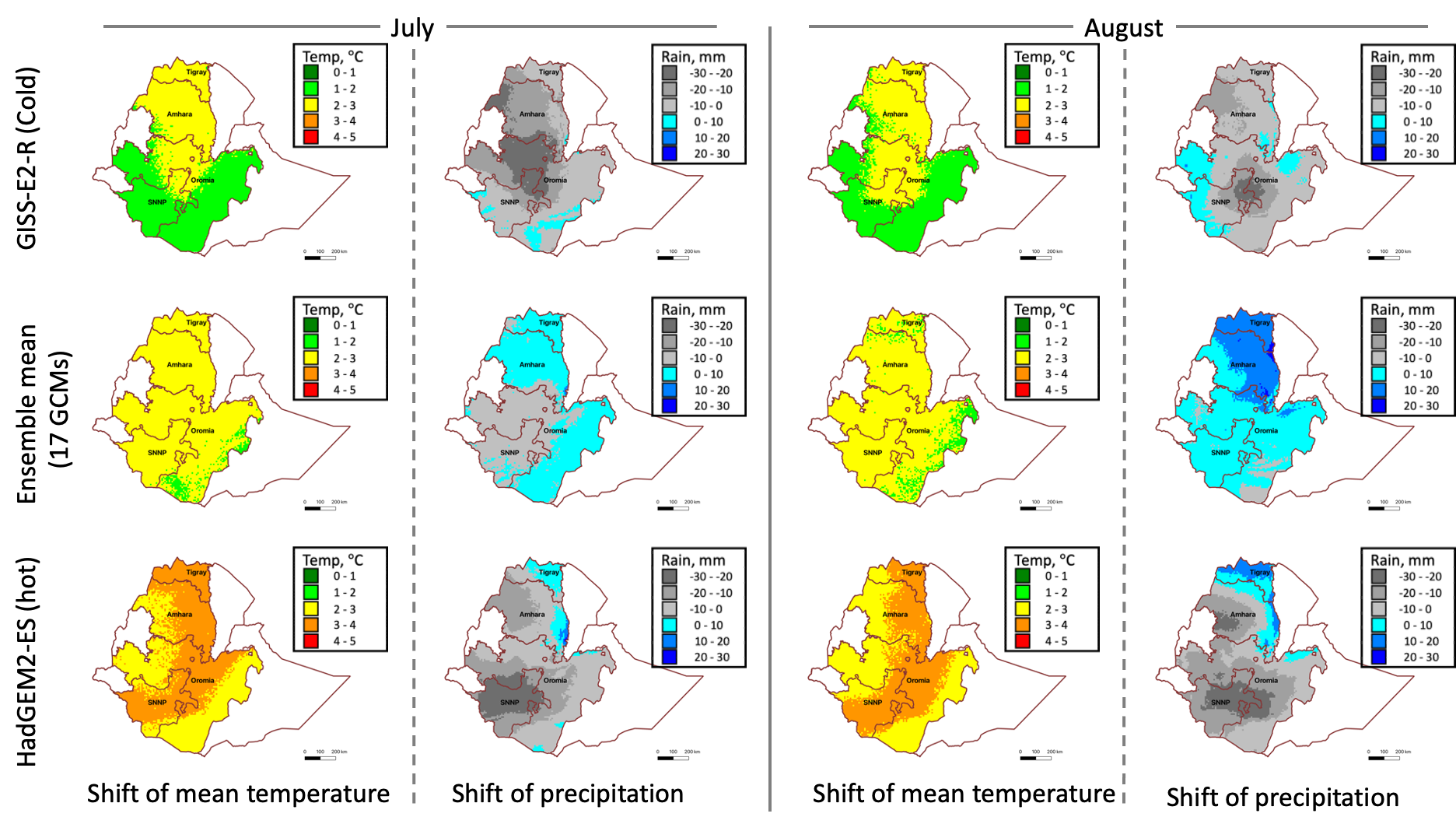


Supplementary Figure S 4. Shifts in mean temperature and precipitation during July and August for Tigray, Oromia, Amhara and SNNP predicted by three future climate projections (2050) compared to 1970-2000 baselines. Maps were generated using software ArcGIS Pro software (https://pro.arcgis.com).

**
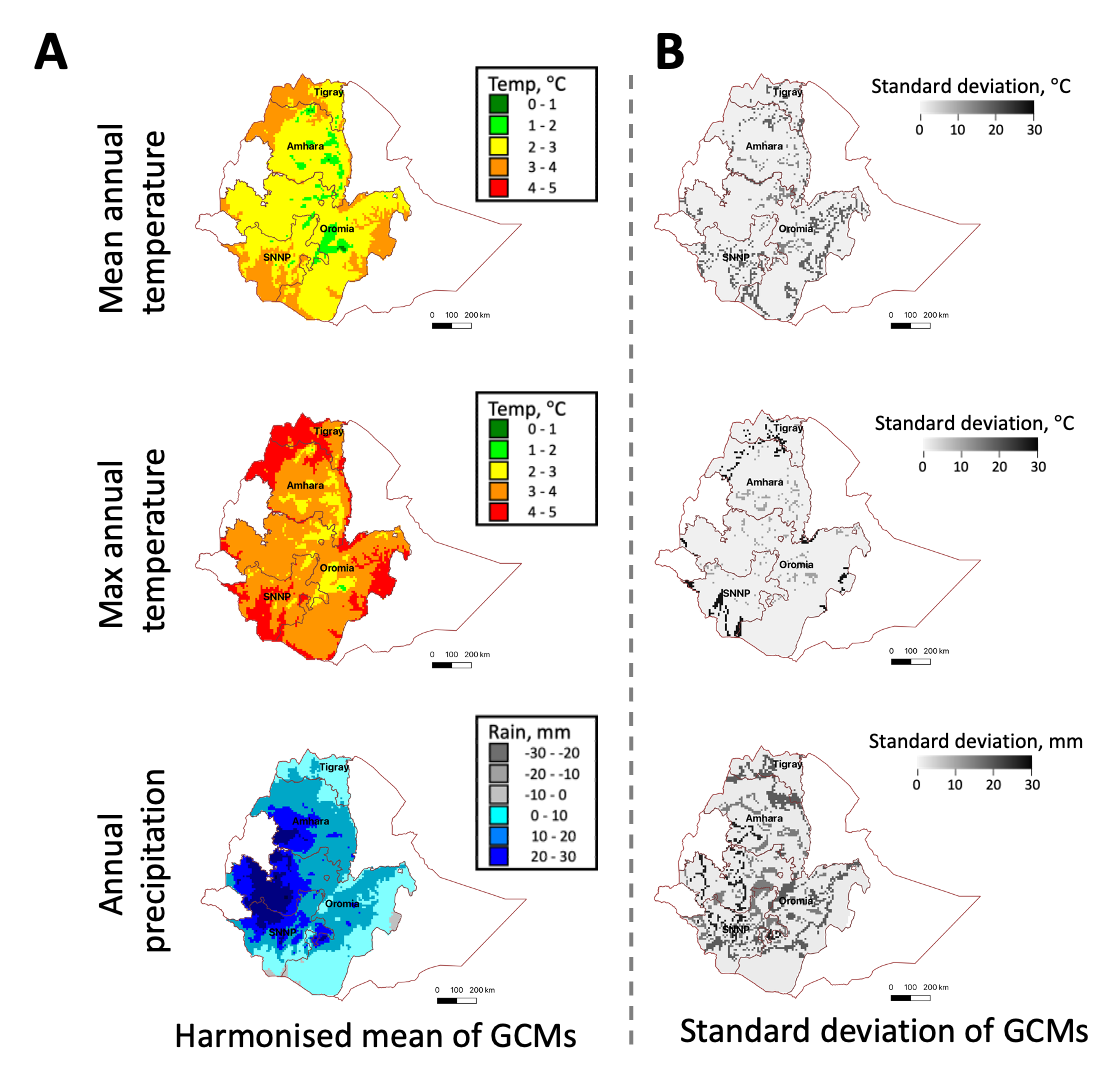
**

Supplementary Figure S 5.Uncertainties in GCMs climate prediction across the region. Harmonic mean of annual mean and maximum temperature and precipitation (A) and Standard deviation among predictions (B) for Tigray, Amhara, Oromia and SNNP areas. Maps were generated using software ArcGIS Pro software (https://pro.arcgis.com).

**
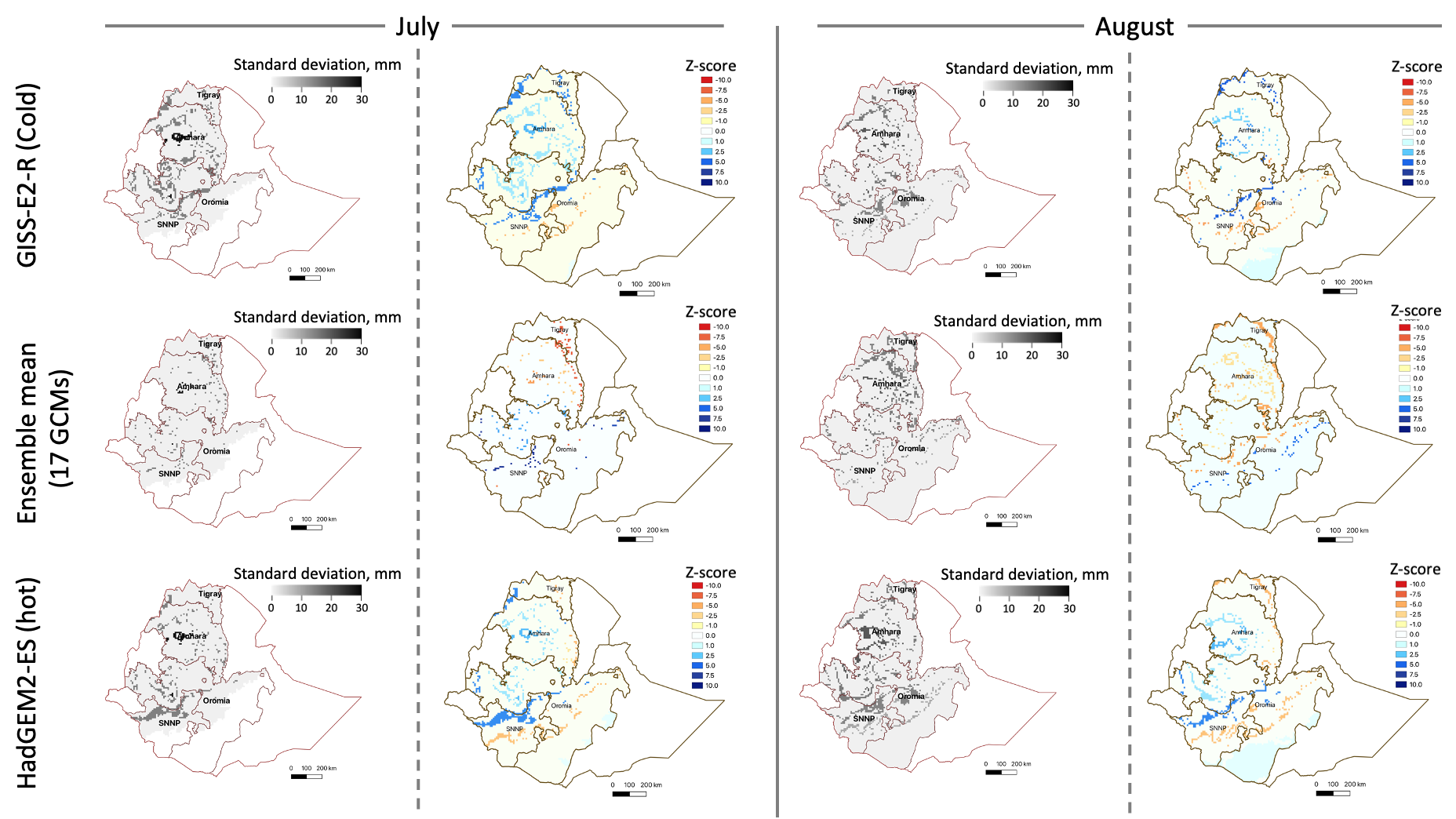
**

Supplementary Figure S 6. Evaluation of uncertainties in precipitation change in 2050. Standard deviation among precipitation predictions with GISS-2E-R, 17 GCMs and HadGEM2-ES models vs current climate data across for Tigray, Amhara, Oromia and SNNP areas. Z-score of precipitation change in the four regions in 2050. Maps were generated using software ArcGIS Pro software (https://pro.arcgis.com).

**
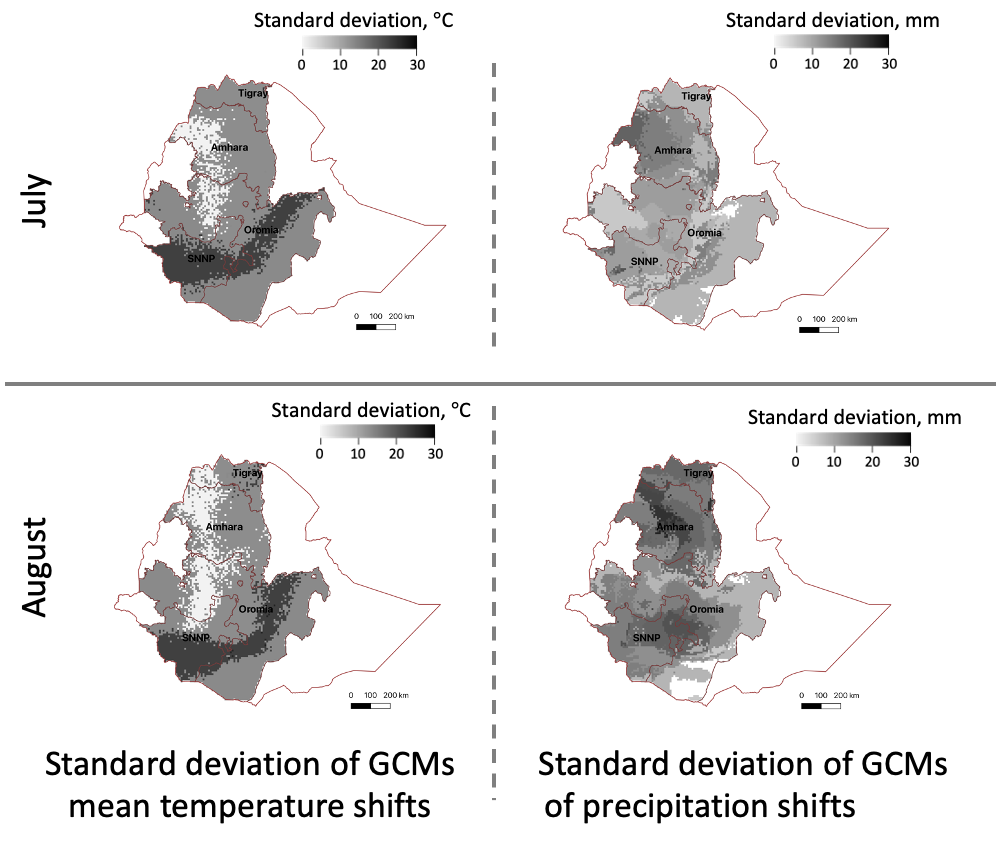
**

Supplementary Figure S 7. Standard deviation among mean temperature shifts and precipitation shifts predictions with GISS-2E-R, 17 GCMs and HadGEM2-ES models for Tigray, Amhara, Oromia and SNNP areas. Maps were generated using software ArcGIS Pro software (https://pro.arcgis.com).

**SUPPLEMENTARY TABLES**

Supplementary Table S 1. Details of global circulation models (GCMs) used in this study showing predicted shifts in annual rainfall and average temperature for land surface between latitudes 30 °N and 30 °S by the 2050s under three scenarios; values are shown as changes from current values. Details of the GCMs used are shown in ^4^. 11

Supplementary Table S 2. Parameters of Bioclimatic factors variables used for Ecocrop modelling of Buffel, Napier and Rhodes grasses in four regions in Ethiopia. 12

Supplementary Table S 3. Results of uncertainty evaluation by Monte Carlo Method are calculated based for the best Buffel, Rhodes and Napier grass combination to maximise productivity on specific areas predicted for different climatic scenarios (GISS-E2-R (Cold), Ensemble mean (17 GCMs), and HadGEM2-ES (hot). 13

Supplementary Table S 4. Areas suitable for Buffel, Napier and Rhodes grass cultivation, under a 15% and 10% land availability scenario. 14

Supplementary Table S 1. Details of global circulation models (GCMs) used in this study showing predicted shifts in annual rainfall and average temperature for land surface between latitudes 30 °N and 30 °S by the 2050s under three scenarios; values are shown as changes from current values. Details of the GCMs used are shown in ^4^.

| **GCM** | **2050s** | |
| --- | --- | --- |
|  | **Annual rainfall (mm)** | **Mean annual temperature (°C)** |
| **GISS-E2-R**  **(NASA Goddard Institute for Space Studies)** | -53 | +1.96 |
| **Ensemble mean**  **(17 GCMs)** | +12 | +2.36 |
| **HadGEM2-ES**  **(UK Met Office Hadley Centre)** | -2 | +2.93 |

Supplementary Table S 2. Parameters of Bioclimatic factors variables used for Ecocrop modelling of Buffel, Napier and Rhodes grasses in four regions in Ethiopia.

| Parameters used in the model | Buffel grass *(Cenchrus ciliaris L)* | Napier grass (*Pennisetum purpureum Schum*.) | Rhodes grass (*Chloris gayana Kunth*) |
| --- | --- | --- | --- |
| Length of growing season | | | |
| Gmin | 120 | 120 | 150 |
| Gmax | 180 | 210 | 210 |
| Gused | 150 | 165 | 180 |
| Temperature variables | | | |
| Ktmp | 0 | -1 | -10 |
| Tmin | 5 | 15 | 5 |
| TOPmn | 22 | 21 | 20 |
| TOPmx | 37 | 40 | 37 |
| Tmax | 42 | 45 | 50 |
| Precipitation variables | | | |
| Rmin | 270 | 850 | 550 |
| ROPmn | 400 | 1000 | 600 |
| ROPmx | 2000 | 1500 | 1500 |
| Rmax | 3500 | 2500 | 2000 |
| Dry period | 6-9 months | 9 months | 6 months |
| Soils | | | |
| Soil pH | 6.5-8.0 | 4.5-8.2 | 5.5-7.5 |
| Soil texture/type | Light, sandy soils and loam, clay loam and red earth soils | Light-heavy soils, clay to clay loam soil | Fertile, well-structured loams and clays soils |

**Lengths of growing season:** Gmin is start of growing season; Gmax is end of growing season; Gused is length of growing season; **Temperature:** Ktmp is absolute temperature that will kill the plant; Tmin is minimum average temperature at which the plant will grow; TOPmn is minimum average temperature at which the plant will grow optimally; TOPmx is maximum average temperature at which the plant will grow optimally; Tmax is maximum average temperature at which the plant will cease to grow; **Precipitation:** Rmin is minimum rainfall (mm) during the growing season; ROPmn is optimal minimum rainfall (mm); ROPmx is optimal maximum rainfall (mm); Rmax is maximum rainfall (mm) during the growing season; **Soil pH:** range of soil pH suitable for plant growth.

Supplementary Table S 3. Results of uncertainty evaluation by Monte Carlo Method are calculated based for the best Buffel, Rhodes and Napier grass combination to maximise productivity on specific areas predicted for different climatic scenarios (GISS-E2-R (Cold), Ensemble mean (17 GCMs), and HadGEM2-ES (hot).

| Scenario | Region | R-factor |
| --- | --- | --- |
| Scenario A, 15% Land is available | Amhara | 4.4 |
|  | Oromia | 6.2 |
|  | SNNP | 2.6 |
|  | Tyger | 4.9 |
| Scenario A, 10% Land is available | Amhara | 3.8 |
|  | Oromia | 5.0 |
|  | SNNP | 2.3 |
|  | Tyger | 4.1 |
| Scenario B, 15% Land is available | Amhara | 3.8 |
|  | Oromia | 4.8 |
|  | SNNP | 2.2 |
|  | Tyger | 4.0 |
| Scenario B, 10% Land is available | Amhara | 3.4 |
|  | Oromia | 4.0 |
|  | SNNP | 2.1 |
|  | Tyger | 3.4 |

Note. The lower the value of R-factor (Rf), the less is uncertainty of prediction.

| Region | Land suitability %, current | | | Current Pasture land suitable for grass cultivation, ha, 15% availability scenario | | |  |  |  |
| --- | --- | --- | --- | --- | --- | --- | --- | --- | --- |
|  | Buffel | Napier | Rhodes | Buffel | Napier | Rhodes |  | | |
| Amhara | 53.8 | 9.4 | 59.8 | 1247394.3 | 218103.6 | 1387760.7 |  |  |  |
| Oromia | 38.3 | 2.9 | 45.8 | 1634883.0 | 122205.2 | 1953208.6 |  |  |  |
| Tigray | 76.7 | 0.0 | 68.3 | 476385.7 | 0 | 424100.4 |  |  |  |
| SNNP | 63.6 | 12.1 | 65.8 | 1010032.9 | 192282.7 | 1045270.5 |  |  |  |
| Region | Land suitability %, future (GISS-E2-R cold) | | | Future (GISS-E2-R cold) Pasture land suitable for grass cultivation, ha, 15% availability scenario | | | Future (GISS-E2-R cold) Pasture land suitable for grass cultivation, ha, 10% availability scenario | | |
|  | Buffel | Napier | Rhodes | Buffel | Napier | Rhodes | \| Buffel \| Napier \| Rhodes \| \| --- \| --- \| --- \| | | |
| Amhara | 67.7 | 16.0 | 80.2 | 1570057.5 | 370828.3 | 1860255.1 | 1046705.0 | 247218.9 | 1240170.1 |
| Oromia | 63.6 | 15.5 | 53.6 | 2716320.7 | 659776.5 | 2287382.9 | 1810880.5 | 439851.0 | 1524921.9 |
| Tigray | 87.0 | 0.0 | 64.1 | 540329.2 | 0 | 398427.7 | 360219.5 | 0 | 265618.4 |
| SNNP | 73.8 | 20.0 | 72.0 | 1172703.7 | 318297.7 | 1143183.3 | 781802.4 | 212198.5 | 762122.2 |
| Region | Land suitability %, future (17GCMs) | | | Future (17GCMs) Pasture land suitable for grass cultivation, ha, 10% availability scenario | | | Future (17 GCMs) Pasture land suitable for grass cultivation, ha, 10% availability scenario | | |
|  | Buffel | Napier | Rhodes | Buffel | Napier | Rhodes | \| Buffel \| Napier \| Rhodes \| \| --- \| --- \| --- \| | | |
| Amhara | 76.3 | 21.3 | 84.1 | 1769607.6 | 494666.9 | 1951322.2 | 1179738.4 | 329777.9 | 1300881.4 |
| Oromia | 65.3 | 18.8 | 59.9 | 2786840.6 | 800449.4 | 2555774.7 | 1857893.7 | 533632.9 | 1703849.8 |
| Tigray | 93.8 | 0.2 | 82.6 | 582776.3 | 1462.4 | 513066.8 | 388517.5 | 974.9 | 342044.5 |
| SNNP | 79.7 | 29.3 | 77.8 | 1265111.9 | 465942.4 | 1236082.0 | 843407.9 | 310628.3 | 824054.7 |
| Region | Land suitability %, future (HadGEM2-ES hot) | | | Future (HadGEM2-ES hot) Pasture land suitable for grass cultivation, ha, 10% availability scenario | | | Future (HadGEM2-ES hot) Pasture land suitable for grass cultivation, ha, 10% availability scenario | | |
|  | Buffel | Napier | Rhodes | Buffel | Napier | Rhodes | \| Buffel \| Napier \| Rhodes \| \| --- \| --- \| --- \| | | |
| Amhara | 68.1 | 25.2 | 89.2 | 1579873.7 | 585080.7 | 2069080.4 | 1053249.1 | 390053.8 | 1379386.9 |
| Oromia | 68.0 | 21.2 | 55.7 | 2902461.5 | 904753.3 | 2377315.3 | 1934974.3 | 603168.8 | 1584876.9 |
| Tigray | 98.4 | 0.0 | 76.8 | 611099.0 | 0 | 476990.3 | 407399.3 | 0 | 317993.5 |
| SNNP | 81.5 | 28.3 | 69.6 | 1294618.2 | 450025.8 | 1106180.2 | 863078.8 | 300017.2 | 737453.5 |

Supplementary Table S 4. Areas suitable for Buffel, Napier and Rhodes grass cultivation, under a 15% and 10% land availability scenario.

**References for Supplementary Material**

1 Berhanu, B., Melesse, A. M. & Seleshi, Y. GIS-based hydrological zones and soil geo-database of Ethiopia. *Catena* **104**, 21-31 (2013).

2 Beck, H. E. *et al.* Publisher Correction: Present and future Köppen-Geiger climate classification maps at 1-km resolution. *Scientific Data* **7**, 1-2 (2020).

3 Peel, M. C., Finlayson, B. L. & McMahon, T. A. Updated world map of the Köppen-Geiger climate classification. *Hydrology and earth system sciences* **11**, 1633-1644 (2007).

4 Jones, P. G. & Thornton, P. K. Representative soil profiles for the Harmonized World Soil Database at different spatial resolutions for agricultural modelling applications. *Agricultural Systems* **139**, 93-99 (2015).
